# Analytical and Biological Factors Associated with Oncogenic Variant Yield in Tumor-Agnostic Plasma ctDNA Profiling

**DOI:** 10.3390/diagnostics16193077

**Published:** 2026-09-22

**Authors:** Stephen George, Lavanya Ranganathan, Yizhen Xiang, Alexander Malt, Shashwat Sahay, Eva-Maria Mayr, Florian Scherer, Hanno Glimm, Stefan Fröhling, Naveed Ishaque, Nicole Pfarr, Damian T. Rieke, Ingeborg Tinhofer

**Affiliations:** 1 German Cancer Consortium (DKTK) Partner Site Berlin and German Cancer Research Center (DKFZ), 69120 Heidelberg, Germany; stephen.george@charite.de (S.G.); damian.rieke@charite.de (D.T.R.); 2 Department of Radiooncology and Radiotherapy, Charité – Universitätsmedizin Berlin, Corporate Member of Freie Universität Berlin and Humboldt-Universität zu Berlin, Charitéplatz 1, 10117 Berlin, Germany; 3 German Cancer Consortium (DKTK) Partner Site Freiburg and German Cancer Research Center (DKFZ), 69120 Heidelberg, Germany; lavanya.ranganathan@uniklinik-freiburg.de (L.R.); florian.scherer@uniklinik-freiburg.de (F.S.); 4 Department of Medicine I, Medical Center – University of Freiburg, Faculty of Medicine, University of Freiburg, 79106 Freiburg, Germany; 5 Berlin Institute of Health at Charité – Universitätsmedizin Berlin, Center of Digital Health, 10117 Berlin, Germany; yizhen.xiang@bih-charite.de (Y.X.); alexander.malt@bih-charite.de (A.M.); shashwat.sahay@bih-charite.de (S.S.); naveed.ishaque@bih-charite.de (N.I.); 6 Institute of Pathology, School of Medicine and Health, Technical University of Munich (TUM), 81675 Munich, Germany; mayr_e@ukw.de (E.-M.M.); nicole.pfarr@tum.de (N.P.); 7 Department of Translational Medical Oncology, National Center for Tumor Diseases (NCT), NCT/University Cancer Center Dresden, a Partnership Between German Cancer Research Center (DKFZ), Faculty of Medicine and University Hospital Carl Gustav Carus, Dresden University of Technology (TUD), and Helmholtz-Zentrum Dresden-Rossendorf, 01307 Dresden, Germany; hanno.glimm@nct-dresden.de; 8 Translational Medical Oncology, Faculty of Medicine and University Hospital Carl Gustav Carus, Dresden University of Technology (TUD), 01307 Dresden, Germany; 9 German Cancer Consortium (DKTK) Partner Site Dresden, 01307 Dresden, Germany; 10 Division of Translational Medical Oncology, German Cancer Research Center (DKFZ), 69120 Heidelberg, Germany; stefan.froehling@nct-heidelberg.de; 11 National Center for Tumor Diseases (NCT), NCT Heidelberg, a Partnership Between DKFZ and Heidelberg University Hospital, 69120 Heidelberg, Germany; 12 German Cancer Consortium (DKTK) Core Center Heidelberg, 69120 Heidelberg, Germany; 13 Institute of Human Genetics, Heidelberg University, 69120 Heidelberg, Germany; 14 Comprehensive Cancer Center, Charité – Universitätsmedizin Berlin, Corporate Member of Freie Universität Berlin and Humboldt-Universität zu Berlin, 10117 Berlin, Germany; 15 Department of Hematology, Oncology and Cancer Immunology, Campus Benjamin Franklin, Charité – Universitätsmedizin Berlin, Corporate Member of Freie Universität Berlin and Humboldt-Universität zu Berlin, 12203 Berlin, Germany; 16 National Center for Tumor Diseases (NCT), NCT Berlin, a Partnership Between DKFZ, Charité Universitätsmedizin, Berlin Institute of Health at Charité (BIH) and Max Delbrück Center (MDC), 10117 Berlin, Germany

**Keywords:** ctDNA burden, liquid biopsy, plasma sequencing, tumor heterogeneity, precision oncology

## Abstract

**Background/Objectives:** Tumor-agnostic plasma ctDNA profiling is used when tumor tissue is unavailable, insufficient, or unrepresentative. We investigated analytical and biological factors associated with oncogenic variant yield. **Methods:** In the prospective EXMASTER study, plasma cfDNA was analyzed using a 265-gene targeted panel. Technical performance was benchmarked using synthetic reference materials and tumor-informed tissue–plasma comparison. Tumor-agnostic analysis used predefined filtering and oncogenic classification. **Results:** Forty patients were included; 34 underwent baseline tumor-agnostic plasma profiling. All expected variants were detected in synthetic reference material at the tested nominal allele fractions, including 0.005 (0.5%). In six selected patients, 32 of 33 evaluable tissue-derived variants within the panel footprint were detected in plasma (97.0% variant-level concordance). Tumor-agnostic profiling retained 603 plasma variants, including 74 oncogenic or likely oncogenic variants. Thirty-one of 34 patients (91.2%) had at least one retained plasma variant, whereas 27 of 34 (79.4%) had at least one oncogenic or likely oncogenic variant. No statistically supported association was observed between tissue-derived MATH score and oncogenic variant yield. The correlation between total retained plasma variant burden and oncogenic variant count was considered descriptive because these measures are mathematically dependent. Increasing filtering stringency reduced the retained plasma call set while preserving most tissue-concordant variants. **Conclusions:** In this exploratory, tumor-heterogeneous cohort, analytical filtering strongly shaped the retained plasma variant set, whereas no statistically supported association was observed between tissue-derived MATH score and oncogenic variant yield. These findings support assay-aware interpretation of plasma sequencing as complementary rather than interchangeable with tissue profiling.

## 1. Introduction

Plasma-based circulating tumor DNA (ctDNA) analysis is increasingly integrated into precision oncology to complement tissue genomic profiling, particularly when tissue is unavailable, insufficient, or potentially unrepresentative [1,2,3,4,5]. However, tissue and plasma sequencing provide non-identical views of tumor genomics. Plasma assays may identify alterations not captured in a single tissue biopsy but may also miss tissue-derived alterations because of variable tumor DNA shedding, treatment exposure, metastatic site, sampling interval, clonal architecture, and analytical sensitivity [4,6,7,8,9,10,11,12,13,14].

A practical challenge for molecular tumor boards is that detection of plasma variants does not automatically translate into findings with established biological or therapeutic relevance. Tumor-agnostic workflows identify variants directly from plasma and therefore require filtering to reduce technical artifacts, germline variants, and clonal hematopoiesis of indeterminate potential (CHIP), followed by oncogenic classification [9,15,16,17]. Thus, the clinically relevant question is not only whether ctDNA can be detected but also which analytical and biological factors are associated with the recovery of oncogenic or potentially therapeutically relevant findings through tumor-agnostic plasma profiling.

Tumor heterogeneity is one proposed driver of the added value of plasma sequencing, yet single-biopsy measures such as mutant-allele tumor heterogeneity (MATH) [18] score may not reflect systemic ctDNA representation across metastatic sites. Within the prospective EXMASTER study, we therefore explored analytical and biological factors associated with variation in the output of tumor-agnostic plasma profiling, focusing on analytical filtering, plasma variant burden, and tissue-derived MATH score in a clinically selected precision-oncology cohort. We first benchmarked technical performance and tumor-informed tissue–plasma concordance and then assessed tumor-agnostic filtering, oncogenic variant yield, tissue–plasma reconciliation of potentially therapeutically relevant findings, and longitudinal ctDNA dynamics. These analyses were exploratory and hypothesis-generating.

## 2. Materials and Methods

### 2.1. Patient Cohort

The prospective EXMASTER study independently analyzed cfDNA linked to tumor tissue sequencing data from the German Cancer Consortium (DKTK) Molecularly Aided Stratification for Tumor Eradication Research (MASTER) program (ClinicalTrials.gov identifier: NCT05852522) [19]. MASTER enrolls patients with advanced malignancies for comprehensive molecular profiling, with emphasis on rare tumor types, rare molecular subtypes, and younger patients. The MASTER program was approved by the Ethics Committee, Medical Faculty Heidelberg (S-266/2011; 5 October 2015) and the Ethics Committee, Charité – Universitätsmedizin Berlin (EA4/075/16; 27 July 2016). Additional ethics approval was obtained from the Ethics Committee, Charité – Universitätsmedizin Berlin, for prospective collection and biobanking of liquid biopsies (EA2/152/10; 22 June 2023) and retrospective molecular analysis (EA2/180/24; 27 September 2024). Written informed consent was obtained from all participants.

Patients were selected from the MASTER cohort based on the availability of plasma samples and the data required for the respective analyses. The study included 40 patients. Of those, 34 underwent baseline tumor-agnostic plasma profiling, and six underwent tumor-informed technical benchmarking (Supplementary Figure S1). Among the 34 baseline tumor-agnostic patients, 31 had evaluable tissue-derived MATH scores, and 29 had tissue TMB data. Two patients lacked matched tumor tissue sequencing; one additional patient had no evaluable MATH value in the analysis dataset, and tissue TMB was unavailable for two additional MATH-evaluable patients. Tissue-derived heterogeneity analyses were restricted to patients with suitable tumor tissue sequencing data, while longitudinal analyses were restricted to patients with serial plasma samples. The cohort included cases in which tissue-based molecular profiling was uninformative or limited. Information on whether the tumor specimen used for MASTER sequencing originated from the primary tumor or a metastatic site, and on its specific anatomical sampling site, was not consistently available across the cohort.

Detailed information on systemic treatment status at the time of plasma sampling, tissue–plasma sampling intervals, systemic therapy occurring between tissue and plasma collection, metastatic-site distribution, and quantitative disease burden was likewise not systematically abstracted in the study dataset and was therefore not available for consistent cohort-level analysis.

Longitudinal clinical response annotations were based on routine clinical and radiographic assessments documented by the treating clinicians; formal RECIST classification was not systematically recorded or verified in the study dataset.

### 2.2. Sample Collection

Baseline blood samples were collected within the Charité Comprehensive Cancer Center molecular tumor board workflow, where feasible, concurrently with tumor tissue sampling. Additional samples were obtained during routine follow-up in selected patients, including at progression when available.

Blood samples were collected in Cell-Free DNA BCT^®^ stabilization tubes (Streck, La Vista, NE, USA) according to the study SOP. The maximum permitted interval between blood collection and first centrifugation was 14 days; in practice, samples were typically processed within 7 days of collection. Exact sample-specific collection-to-first-centrifugation intervals were recorded operationally but were not treated as a prespecified study variable or included in the present analyses.

### 2.3. Plasma Sequencing and Analytical Framework

Plasma cfDNA was analyzed using an ExTarget targeted sequencing panel covering 265 cancer-relevant genes (~540 kb; gene list in Supplementary Table S1). A related ExTARGET targeted ctDNA platform has been described by Ranganathan et al. [20]. Library preparation and sequencing were performed in two batches corresponding to tumor-informed technical benchmarking and tumor-agnostic analyses. Sequencing data were processed using unique molecular identifier-aware pipelines with molecular error suppression and predefined quality filters. Detailed laboratory and bioinformatic procedures are provided in the Supplementary Information.

Sequencing quality was assessed using MultiQC summaries incorporating mosdepth coverage, fgbio UMI-family metrics, Picard HsMetrics and target-region metrics, insert-size metrics, and samtools alignment statistics. For the 34 baseline tumor-agnostic plasma samples, median target coverage was 2960× (range, 31–4629×) after consensus processing permitting UMI families of size ≥ 1 and 1671× (range, 11–2725×) after restricting consensus generation to UMI families of size ≥ 3 (Supplementary Table S2). Picard HsMetrics showed a median usable-bases-on-target percentage of 34.6% (range, 0.4–36.7%), and the median insert size was 166 bp (range, 159–178 bp). One sample showed particularly low coverage (31× and 11×, respectively); excluding this sample descriptively, the next-lowest coverage values were 692× and 308×. This sample was retained because no post hoc sequencing-QC exclusion criterion was applied. A sequencing depth of 2500× was used as a target rather than a formal exclusion threshold, and no plasma samples were excluded from analysis on the basis of sequencing coverage or other sequencing QC metrics. Plasma libraries were generated from 50 ng cfDNA input. Patient-specific total cfDNA extraction yield was not systematically available. Conventional duplication rate, a separate pre-consensus raw-depth summary, and a separately defined unique-molecular-depth metric were not generated as cohort-level reporting metrics within this UMI-consensus workflow and were not retrospectively reconstructed.

Two analytical approaches were applied. Tumor-informed analysis queried plasma for variants previously identified in tumor tissue. For tumor-informed benchmarking, six patients were selected before plasma processing because matched tumor sequencing had identified one or more variants within the ExTarget panel footprint; selection therefore did not depend on plasma ctDNA positivity or subsequent plasma detection results. Tumor-agnostic analysis identified variants de novo from plasma and applied predefined candidate filtering configurations followed by oncogenicity classification.

Variants detected in matched buffy coat DNA were excluded to remove germline and CHIP-derived variants. Potential germline cancer-predisposition variants were therefore excluded when detected in matched buffy coat DNA and did not contribute to the tumor-agnostic plasma calls. Additional filtering included an exclusion list generated from healthy donor plasma to reduce recurrent technical artifacts. Because low-allele-fraction CHIP variants may escape detection in buffy coat sequencing, tumor-agnostic analyses also excluded variants in canonical CHIP-associated genes, including *DNMT3A*, *TET2*, *ASXL1*, *JAK2*, *PPM1D*, *SF3B1*, *SRSF2*, and *U2AF1*. *TP53* was handled separately because of its dual role in tumorigenesis and clonal hematopoiesis: variants were retained when consistent with oncogenic driver events, such as hotspot or truncating alterations, whereas low-confidence variants lacking such features were excluded.

### 2.4. Variant Classification and Endpoints

Plasma variants were interpreted using a structured oncogenic classification framework incorporating publicly curated databases, including OncoKB (data release v6.0) and ClinVar, and following consensus recommendations for somatic variant interpretation [21,22,23]. Variants were categorized as oncogenic, likely oncogenic, benign, likely benign, or of uncertain significance. For analysis, oncogenic and likely oncogenic variants were grouped together. Oncogenic classification was considered separately from therapeutic annotation. Tissue-based therapeutic findings reflected recommendations documented by the molecular tumor board. For plasma-only variants classified as oncogenic or likely oncogenic, potential therapeutic associations were assigned retrospectively for research purposes through targeted searches of the published literature and publicly accessible biomarker, variant, and clinical-trial resources. Searches considered the specific variant or altered gene, relevant drug or drug class, and the patient’s tumor type. Supporting evidence included PubMed-indexed literature, ClinicalTrials.gov records, and publicly available variant or biomarker databases such as ClinVar where relevant. All candidate plasma-only therapeutic associations were subsequently re-evaluated using the ESMO Scale for Clinical Actionability of molecular Targets (ESCAT), considering the specific molecular alteration, therapy or drug class, tumor type, and available clinical or preclinical evidence. ESCAT tiers were assigned retrospectively according to the published ESCAT framework, as detailed in the Supplementary Information, and cross-tumor, pathway-level, or preclinical extrapolation was explicitly identified where applicable in Supplementary Table S3. ESCAT was used to grade the strength of evidence among retained associations rather than as a prespecified minimum-tier inclusion threshold. Associations that were not supported after formal evidence review were not retained in the final therapeutic-association analysis. ESCAT was used as the formal therapeutic evidence framework; additional OncoKB therapeutic levels or AMP tiers were not assigned. Annotations and ESCAT assessment were performed by a single investigator and were not independently adjudicated. Plasma-only associations were not reviewed by the molecular tumor board and did not guide clinical decision-making.

For clarity, four non-equivalent levels of interpretation were distinguished: (1) analytically retained plasma variants, defined as variants remaining after tumor-agnostic filtering; (2) oncogenic or likely oncogenic variants, defined according to the classification framework above; (3) variants with retrospectively assigned potential therapeutic associations supported by post hoc ESCAT assessment, which were annotated for research purposes only; and (4) clinically actionable findings, reserved for variants supported by documented molecular tumor board recommendations or clinical intervention.

Intratumoral heterogeneity was quantified using the MATH score, reflecting dispersion of variant allele fractions in tumor tissue [18]. The score was calculated from somatic variants after tissue-level quality filtering and evaluated in relation to plasma-only variant burden and oncogenic variant yield.

In tumor-informed analyses, ctDNA detection was defined as the identification of at least one tissue-derived variant in plasma. Concordance was assessed at the variant level as the proportion of tissue variants detected in plasma, restricted to variants within the plasma panel footprint. Variants detected in both matched tissue and plasma were classified as tissue–plasma concordant. Variants detected only in tissue were classified as tissue-only, and variants detected only in plasma were classified as plasma-only. In tumor-agnostic analyses, patient-level analytical detection was defined as the presence of at least one retained somatic plasma variant considered compatible with tumor origin after filtering. This endpoint was distinct from oncogenic positivity, which required at least one retained variant classified as oncogenic or likely oncogenic. Retained plasma variant burden was defined as the number of retained plasma variants, and oncogenic variant count as the number of retained variants classified as oncogenic or likely oncogenic.

### 2.5. Statistical Analysis

Statistical analyses were performed in R version 4.5.2. Continuous variables are reported as medians with ranges or interquartile ranges. As data were not normally distributed, non-parametric methods were used. Associations were assessed using Spearman’s rank correlation. Log10 transformation was applied for visualization where appropriate. Analyses were exploratory and primarily descriptive. Given the exploratory, hypothesis-generating design, no formal sample-size calculation was performed. Given the modest sample size and tumor heterogeneity, multivariable adjustment for tumor type or other clinical determinants of ctDNA shedding was not performed. Each analysis included only patients for whom the required data were available; missing values were not estimated or filled in. The number of patients included is reported for each analysis. Where statistical tests were performed, they were two-sided. Because analyses were exploratory and hypothesis-generating, no adjustment for multiple comparisons was performed; *p* values are therefore reported descriptively and should be interpreted as nominal. Spearman correlation coefficients (ρ), corresponding *p* values, 95% confidence intervals, and the number of evaluable patients were reported for correlation analyses. Confidence intervals for Spearman ρ were estimated using a paired nonparametric percentile bootstrap with 10,000 resamples. Exact 95% confidence intervals for binomial proportions were calculated using the Clopper–Pearson method. Exploratory univariable negative-binomial regression models with a log link were fitted as sensitivity analyses for the count endpoints. Results are reported as incidence rate ratios (IRRs) with 95% Wald confidence intervals.

## 3. Results

### 3.1. Cohort and Analytical Framework

A total of 40 patients were included, of whom 38 had matched tumor tissue sequencing available. At the time point of plasma sampling, 39 of 40 patients (98%) had metastatic disease, while one patient (2%) had non-metastatic disease. Prior tumor tissue profiling included whole-exome sequencing, whole-genome sequencing, and, in a subset, large targeted panel sequencing. The median age was 48.5 years, and 22 of 40 patients (55%) were male. Head and neck cancers were the most common tumor entity group (17/40, 42%), followed by colorectal cancers (7/40, 18%) and non-colorectal gastrointestinal cancers (4/40, 10%) (Table 1).

Six patients were included in a tumor-informed cohort to assess plasma variant detection performance, while 34 patients underwent baseline tumor-agnostic plasma profiling. In addition, plasma from healthy donors (*n* = 8) and synthetic ctDNA reference materials were processed to characterize background signal and support technical benchmarking. Exploratory longitudinal analyses included tumor-informed tracking in five patients and tumor-agnostic profiling in six patients, including one patient without matched tissue sequencing.

### 3.2. Technical Benchmarking and Tumor-Informed Detection

The EXMASTER workflow was technically benchmarked using synthetic ctDNA reference material containing 12 predefined variants covered by the ExTarget panel. Reference material was analyzed at nominal allele fractions of 0.025 (2.5%), 0.01 (1.0%), and 0.005 (0.5%), alongside a wild-type control. All expected variants were detected at each tested allele fraction, including 0.005 (0.5%), and observed allele fractions closely matched nominal input values (Figure 1A); this experiment was used for technical benchmarking and was not designed to establish a formal assay limit of detection. No variants passed final filtering in the wild-type control.

To benchmark tumor-informed variant detection, six patients with matched tumor tissue sequencing were evaluated. Among the 33 evaluable tissue-derived variants within the ExTarget panel footprint, 32 were detected in plasma, corresponding to a variant-level concordance of 97.0% (exact 95% CI, 84.2% to 99.9%). The proportion of evaluable tissue-derived variants detected in plasma per patient ranged from 88% to 100%. Patient-level characteristics and tumor-informed variant detection results are provided in Supplementary Table S4. Plasma allele fractions were consistently lower than tissue allele fractions (Figure 1B). Within this selected six-patient cohort, these results showed high tumor-informed concordance for predefined tissue-derived variants within the panel footprint. This selected variant-level benchmark does not estimate patient-level clinical sensitivity or general assay performance in an unselected population.

### 3.3. Tumor-Agnostic Filtering Strategy

Baseline plasma samples from 34 patients were first analyzed in a blinded tumor-agnostic manner, with variants identified directly from plasma without using tissue-derived sequencing information. Matched tissue data were then incorporated post hoc to determine which plasma variants were concordant with tissue-derived alterations within the panel footprint. This allowed evaluation of tissue-concordant tumor-derived signal without using tissue information for initial tumor-agnostic variant calling.

Three predefined candidate filtering configurations of increasing stringency were evaluated using tissue-detected variants within the panel footprint as a reference. Detailed parameters are provided in the Supplementary Information. Increasing filtering stringency substantially reduced the retained plasma call set while preserving most tissue-concordant variants. Among 28 patients with paired UMI1 and UMI3 processed datasets, Tier 1 retained 2392 cfDNA variant calls, Tier 2 retained 1070, and Tier 3 retained 501 (Supplementary Table S5). Separately, among 21 patients with panel-evaluable tissue DNA variants, Tier 1 retained 58 tissue-concordant variants, Tier 2 retained 57, and Tier 3 retained 53, corresponding to 91.4% retention under Tier 3 relative to Tier 1. Tier 3 was selected for downstream tumor-agnostic analyses based on this within-cohort balance between a reduction in the retained plasma call set and preservation of tissue-concordant signal.

### 3.4. Tumor-Agnostic Plasma Variant Yield

In the complete 34-patient tumor-agnostic baseline cohort, application of the selected Tier 3 filtering configuration retained 603 plasma variants (Supplementary Table S6). The median number of retained variants per patient was 18.5, with substantial inter-patient variability (range 0–48). Of the retained variants, 74 were classified as oncogenic or likely oncogenic (Supplementary Table S6), corresponding to 12.3% of the retained variant set and a median of two oncogenic or likely oncogenic variants per patient (range: 0–10). At the patient level, 31 of 34 patients (91.2%) had at least one retained plasma variant, whereas 27 of 34 patients (79.4%) had at least one variant classified as oncogenic or likely oncogenic. Thus, patient-level analytical detection and oncogenic positivity represented distinct endpoints.

### 3.5. Associations with Oncogenic Variant Yield

We next examined whether tissue-derived heterogeneity or quantitative plasma metrics were associated with oncogenic tumor-agnostic plasma output. In this exploratory cohort, no statistically supported association was observed between tissue-derived MATH score and either the total number of plasma-only variants absent from matched tissue (Figure 2A; *n* = 31; Spearman ρ = −0.05; 95% CI, −0.41 to 0.34; *p* = 0.79) or the number of oncogenic plasma-only variants (Figure 2B; *n* = 31; Spearman ρ = 0.06; 95% CI, −0.33 to 0.44; *p* = 0.76). No statistically supported association was observed between tissue tumor mutational burden (TMB) and the number of oncogenic plasma variants (Figure 2C; *n* = 29; Spearman ρ = 0.13; 95% CI, −0.26 to 0.52; *p* = 0.49). No statistically supported association was observed between the median allele fraction of retained plasma variants and the number of oncogenic plasma variants (Figure 2D; *n* = 31; Spearman ρ = 0.05; 95% CI, −0.32 to 0.40; *p* = 0.81).

No statistically supported association was observed between the total number of retained plasma variants and the fraction classified as oncogenic or likely oncogenic (Figure 2E; *n* = 31; Spearman ρ = −0.25; 95% CI, −0.64 to 0.18; *p* = 0.18). As expected, the total number of retained variants increased with the absolute number of oncogenic or likely oncogenic variants (Figure 2F; *n* = 34; Spearman ρ = 0.63; 95% CI, 0.33 to 0.83; *p* < 0.001). Because oncogenic variants contribute to the total retained variant count, this part–whole relationship was considered descriptive and was not interpreted as evidence of an independent biological association. In this exploratory cohort, no statistically supported associations were observed between oncogenic variant yield and tissue-derived MATH score, tissue TMB, or the allele-fraction-based plasma signal metric. Exploratory negative-binomial sensitivity analyses of the count endpoints in Figure 2A–D yielded results consistent with the primary Spearman analyses, with all 95% confidence intervals for the IRRs including 1 (Supplementary Table S7).

### 3.6. Reconciliation of Tissue-Based Therapeutic Findings and Retrospectively Annotated Plasma-Only Associations

Tissue alterations associated with molecular tumor board recommendations were compared with tumor-agnostic plasma findings using strict variant-level concordance (Figure 3A). Seventeen tissue-derived alterations were technically assessable within the ctDNA panel footprint. Nine of 17 alterations (53%) were detected in plasma, whereas eight were not detected. Detected tissue-concordant alterations involved events in *PIK3CA*, *FGFR2*, *HRAS*, *NRAS*, *PTEN*, *ERBB2*, *BRCA2*, and *GNAS*.

Tumor-agnostic profiling identified 15 plasma-only patient-variant findings with retrospectively assigned potential therapeutic associations in 9 of 34 patients (26%) (Figure 3B), representing 13 distinct plasma-only variants. Following formal post hoc ESCAT assessment, the 13 distinct variant–therapy associations were classified as ESCAT II-B (*n* = 2), III-A (*n* = 5), IV-A (*n* = 4), or V (*n* = 2), demonstrating substantial variation in the strength of supporting therapeutic evidence. These findings involved genes and pathways associated with DNA damage response, receptor tyrosine kinase signaling, PI3K–AKT–mTOR signaling, and Notch signaling. Additional patient-level information on tumor entity, therapeutic rationale, evidence context, and ESCAT tier for all therapeutically annotated tissue and plasma findings is provided in Supplementary Table S3. The plasma-only associations were generated retrospectively for research purposes, were not reviewed by the molecular tumor board, and did not guide clinical treatment decisions.

### 3.7. Exploratory Longitudinal ctDNA Dynamics

In exploratory longitudinal analyses, serial plasma samples from six patients were analyzed across three clinically aligned timepoints. Five patients had matched tumor tissue sequencing data enabling tumor-informed tracking, while one additional patient was analyzed only using the tumor-agnostic framework. Tumor-informed ctDNA was detected in four of five patients; among these four patients, 50–100% of evaluable oncogenic tissue-derived variants were detected in at least one plasma sample.

Variant allele fractions showed patient-specific changes over time that were broadly consistent with the documented clinical course (Figure 4A). In individual patients, allele fractions increased during progression or decreased following response. In one patient, tumor-informed variants reappeared approximately two months before radiographic progression.

Tumor-agnostic longitudinal profiling showed patient-specific patterns of variant emergence, persistence, and disappearance (Figure 4B). Some patients maintained relatively high variant counts across timepoints, whereas others developed detectable tumor-agnostic plasma variants during progression. Tumor-agnostic analysis also identified variants emerging during follow-up that were not included in the baseline tissue-informed variant set. Although treatment-specific resistance mechanisms could not be established, longitudinally detected alterations involved genes and pathways previously associated with therapeutic resistance in selected tumor contexts, including *ESR1*, *AR*, and *NF1*, as well as MAPK, PI3K–AKT–mTOR, and Notch signaling. These observations were descriptive and hypothesis-generating.

## 4. Discussion

This study addresses a practical interpretation challenge in molecular diagnostics and precision oncology: which analytical and biological factors are associated with retained plasma variant burden and oncogenic or likely oncogenic variant yield from tumor-agnostic plasma ctDNA profiling. In this clinically selected, tumor-heterogeneous cohort, the composition and yield of tumor-agnostic plasma findings varied substantially with analytical filtering stringency, whereas no statistically supported association was observed between tissue-derived MATH score and oncogenic variant yield. These exploratory findings support a distinction between tumor-informed detection of predefined variants and de novo tumor-agnostic plasma profiling, in which analytical retention, oncogenic classification, and potential therapeutic relevance represent distinct levels of interpretation.

Tumor-informed analysis provided a within-study benchmark for variant detection because plasma was queried for variants already identified in matched tumor tissue. In the selected six-patient cohort, tissue–plasma concordance was high for evaluable tissue-derived variants within the ExTarget panel footprint, consistent with prior studies showing reliable detection of known tumor-derived variants when ctDNA is present at sufficient levels [12,24,25,26]. However, this high tumor-informed concordance did not imply equivalent performance under tumor-agnostic conditions. Under tumor-agnostic conditions, variants must be identified de novo and distinguished from sequencing artifacts, germline variation, clonal hematopoiesis, and low-confidence background signal [9,15,16,17]. Thus, tumor-informed detection and de novo tumor-agnostic variant identification represent related but distinct analytical settings. For broader platform-level context, Ranganathan et al. [20] recently reported a related ExTARGET targeted ctDNA approach applied to 157 plasma specimens from 57 patients with advanced cancers, with tumor variants detected in 96% of baseline samples. This larger study provides external context for the broader ExTARGET approach but does not independently validate the specific Tier 3 tumor-agnostic filtering configuration used in the present study.

Filtering stringency materially influenced tumor-agnostic interpretation. Increasing stringency reduced the retained plasma call set while preserving most tissue-concordant variants within the panel footprint, illustrating the trade-off between analytical sensitivity and confidence when matched tumor information is unavailable. However, only 12.3% of retained variants were classified as oncogenic or likely oncogenic, emphasizing that variant detection alone is insufficient for clinical interpretation. Importantly, classification as oncogenic or likely oncogenic does not by itself establish therapeutic actionability, which additionally depends on tumor type, variant-specific evidence, treatment setting, and available therapies. These findings underscore that plasma results should be interpreted in relation to assay design, filtering thresholds, CHIP mitigation, tumor type, disease distribution, and the timing and site of tissue sampling.

Plasma sequencing is often viewed as a way to sample tumor heterogeneity beyond a single tissue biopsy. However, multi-region and autopsy studies indicate that ctDNA represents this heterogeneity selectively rather than comprehensively [27,28]. Plasma preferentially captures truncal, clonally prevalent, or widely distributed alterations, whereas regionally restricted variants or lesions with low DNA shedding may be underrepresented [27,28,29,30,31,32]. Thus, plasma reflects an aggregate systemic signal shaped by clonal architecture, lesion distribution, anatomical site, treatment exposure, and tumor DNA shedding.

In this context, the absence of a statistically supported association between MATH score and oncogenic plasma output may reflect limitations of single-biopsy heterogeneity metrics. The MATH score captures allelic dispersion within the sampled tumor specimen [18] but does not measure spatial heterogeneity across metastatic sites or the relative contribution of individual lesions to circulating DNA. Given the modest cohort size, this null result should be interpreted cautiously and does not exclude a relationship between tumor heterogeneity and plasma ctDNA findings. Therefore, our findings should not be interpreted as evidence that tumor heterogeneity is irrelevant to ctDNA profiles. Rather, the findings are compatible with plasma variant yield being influenced by biological factors not captured by a single-biopsy MATH score, including systemic ctDNA availability and lesion-level shedding.

Comparison with therapeutically relevant tissue-based findings further illustrated that tissue and plasma profiling provide complementary but non-interchangeable molecular information. Some assessable tissue-derived alterations were detected in plasma, whereas others were not, a pattern consistent with known influences on ctDNA detectability, including tumor burden, metastatic site, timing of sampling, and treatment exposure [11,12,13]. Conversely, tumor-agnostic plasma profiling identified additional plasma-only variants for which potential therapeutic associations could be assigned retrospectively. Formal post hoc ESCAT assessment demonstrated heterogeneous levels of supporting evidence across these associations, ranging from ESCAT II-B to V. Such plasma-only findings may reflect unsampled tumor regions, temporal evolution after tissue acquisition, or subclonal dynamics but may also arise from biological or technical background [7,9,27]. Their presence therefore should not be equated with a validated therapeutic biomarker without variant- and tumor-specific clinical interpretation. Interpretation of such findings requires consideration of assay scope, variant confidence, CHIP mitigation, tumor type, and clinical context.

In exploratory longitudinal analyses, tumor-informed and tumor-agnostic plasma profiling showed patient-specific molecular changes over time. In individual patients, tumor-informed allele fractions changed in patterns broadly consistent with the clinical course, while tumor-agnostic profiling identified variants that were not included in the baseline tissue-informed target set. These observations suggest that serial tumor-agnostic ctDNA profiling may complement predefined tumor-informed tracking by identifying variants emerging during follow-up. However, the longitudinal subset was small, and the data did not establish treatment-specific resistance mechanisms or validate lead-time performance.

Several limitations should be considered. The cohort was modest and tumor-heterogeneous, limiting statistical power and precluding robust tumor-type-specific conclusions. Because tumor type, disease burden, metastatic distribution, treatment exposure, and anatomical site may influence ctDNA shedding, this heterogeneity may also have obscured or confounded associations between tissue-derived MATH or TMB and plasma-derived endpoints. The targeted mutation-focused panel did not comprehensively assess copy-number alterations, fusions, expression biomarkers, or variants outside the panel footprint. Stringent filtering may have reduced sensitivity for very-low-allele-fraction variants, and CHIP mitigation may have removed true tumor-derived variants in selected contexts. Although the three candidate filtering configurations were defined before comparison of tissue–plasma concordance and retained plasma variant burden, Tier 3 was selected based on its observed performance within this cohort and has not been independently evaluated in an external dataset. The observed balance between reduction in the retained plasma call set and preservation of tissue-concordant variants may therefore be optimistic and requires independent validation. The MATH score was derived from single tumor samples rather than multi-region or metastatic-site profiling. The TMB analysis was restricted to patients with available tissue TMB data and should be regarded as descriptive and hypothesis-generating. Moreover, the observed association between total retained variant count and oncogenic variant count is mathematically non-independent because oncogenic variants contribute to the total retained variant count and should therefore be interpreted descriptively. The findings are specific to the ExTarget panel, sequencing workflow, and filtering strategy used here and should not be generalized directly to other ctDNA assays. The technical benchmarking experiments were not designed as a formal analytical validation study and therefore do not establish a definitive assay limit of detection. Because plasma-negative results can reflect limited tumor DNA shedding, assay coverage, or analytical sensitivity, the absence of a detected plasma alteration should not be interpreted as confirmation of wild-type status. Matched buffy coat sequencing enabled exclusion of potential germline variants from the tumor-agnostic plasma calls, but the study was not designed or validated for germline testing. Available clinical documentation did not consistently identify whether the tissue specimen used for MASTER sequencing originated from a primary tumor or metastatic site, or its specific anatomical sampling location, precluding reliable site-specific tissue–plasma concordance analysis. Tissue–plasma sampling intervals and intervening systemic therapy were likewise not systematically available for cohort-level analysis, limiting our ability to distinguish temporal or treatment-related biological discordance from analytical non-detection.

Taken together, these findings support an assay-aware and clinically contextualized view of tumor-agnostic plasma sequencing. In the selected six-patient benchmark cohort, tumor-informed analysis showed high detection of predefined tissue-derived variants within the panel footprint, whereas the composition and interpretability of de novo tumor-agnostic findings depended strongly on analytical filtering. Tissue sequencing provides detailed characterization of sampled lesions, whereas plasma sequencing can provide complementary information on tumor-derived DNA in circulation and may support longitudinal monitoring. Plasma findings, including negative results, should be interpreted in relation to retained plasma variant burden, assay design and filtering strategy, CHIP mitigation, tumor type, and tissue-sampling context rather than being treated as direct evidence of the presence or absence of actionable alterations. Accordingly, the present findings are consistent with plasma profiling serving as a complementary, rather than interchangeable, component of integrated molecular diagnostics and precision oncology.

## Figures and Tables

**Figure 1:**
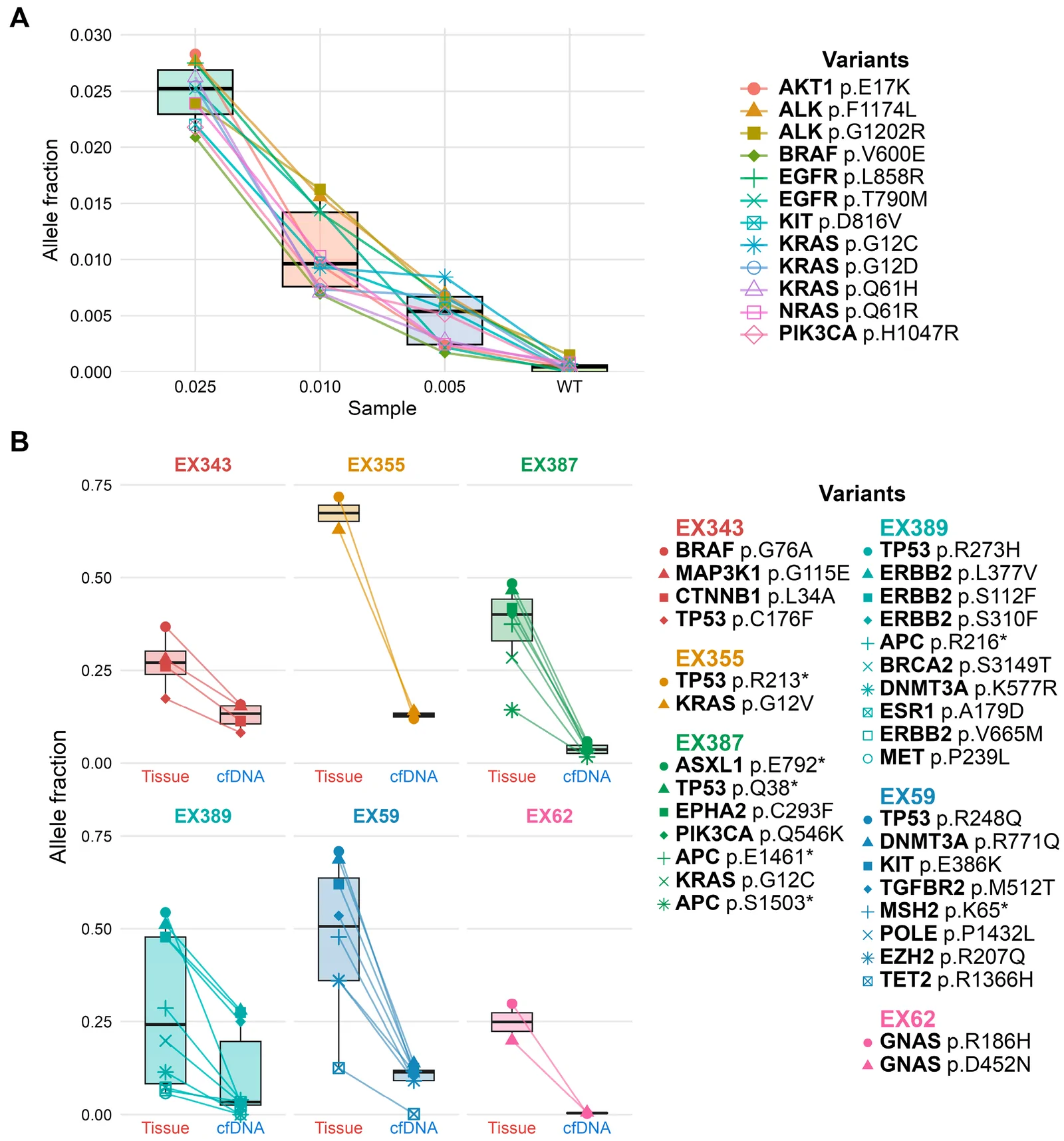
Technical benchmarking of the EXMASTER plasma sequencing workflow and tumor-informed variant detection: (**A**) Observed allele fractions of 12 predefined variants in Seraseq™ ctDNA Mutation Mix reference material analyzed at nominal allele fractions of 0.025 (2.5%), 0.01 (1.0%), and 0.005 (0.5%), with a wild-type control. All expected variants were detected at each tested allele fraction. This technical benchmarking experiment was not designed to establish a formal assay limit of detection. (**B**) Variant allele fractions of matched somatic variants detected in tumor tissue and plasma cfDNA using the ExTarget panel. Lines connect identical variants detected in paired samples. Overall, 32 of 33 evaluable tissue-derived variants within the ExTarget panel footprint were detected in plasma (97.0%; exact 95% CI, 84.2% to 99.9%).

**Figure 2:**
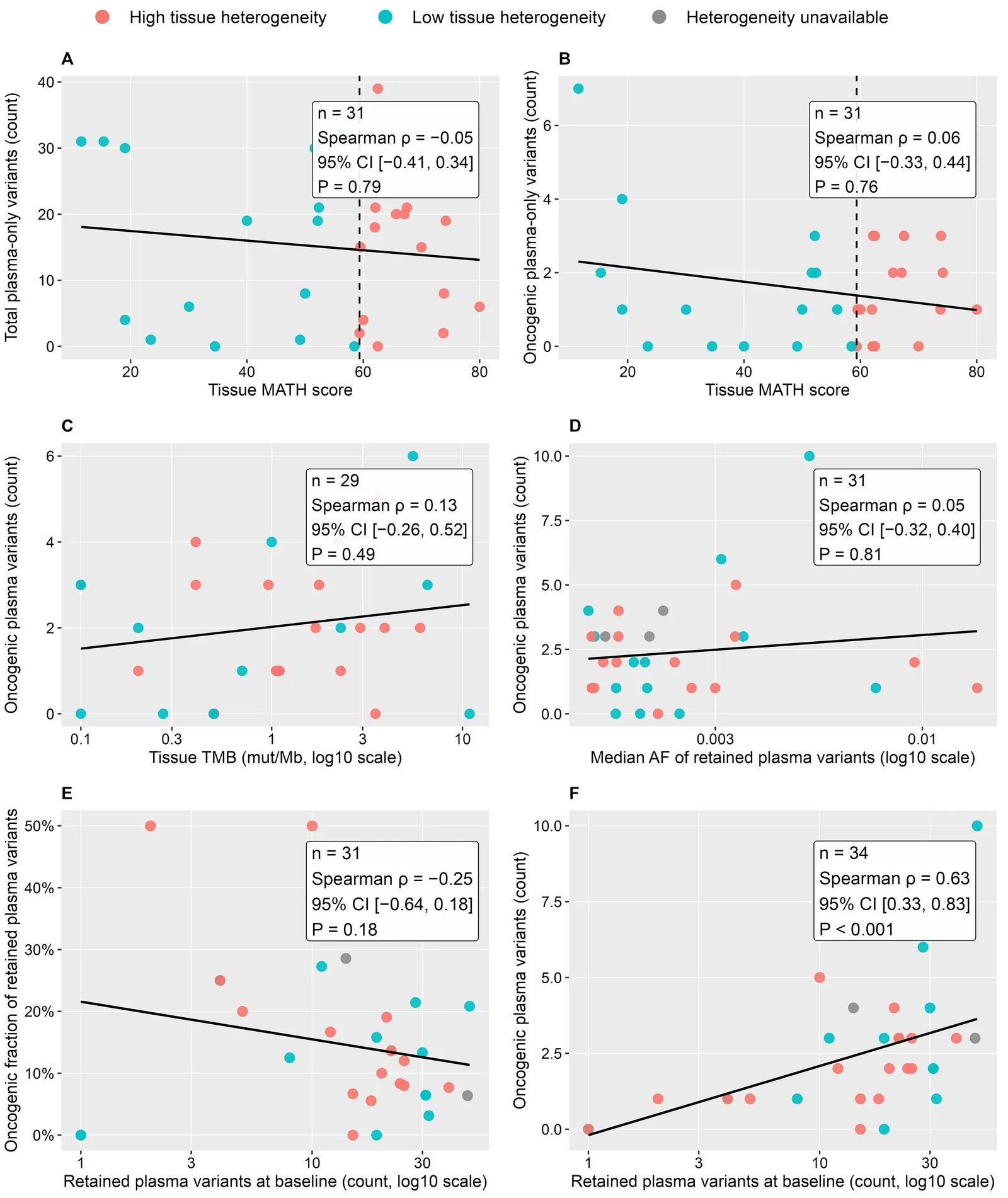
Exploratory associations between tissue- and plasma-derived metrics and oncogenic variant yield under tumor-agnostic plasma profiling. Spearman rank correlations were used to assess associations between (**A**) tissue-derived MATH score and total plasma-only variant count, (**B**) tissue-derived MATH score and oncogenic plasma-only variant count, (**C**) tissue tumor mutational burden and oncogenic plasma variant count, (**D**) median allele fraction of retained plasma variants and oncogenic plasma variant count, (**E**) total retained plasma variant burden and the fraction of retained variants classified as oncogenic or likely oncogenic, and (**F**) total retained plasma variant burden and oncogenic plasma variant count. Spearman correlation coefficients (ρ), 95% confidence intervals, corresponding *p* values, and numbers of evaluable patients (*n*) are shown for each analysis. Confidence intervals were estimated using a paired nonparametric percentile bootstrap with 10,000 resamples. The number of evaluable patients varied according to the availability of the variables required for each analysis. Analyses were exploratory, and *p* values were not adjusted for multiple comparisons. Solid black lines indicate fitted linear regression lines for visualization. Dashed vertical lines in panels (**A**,**B**) indicate the median MATH score used to define the high- and low-tissue-heterogeneity groups.

**Figure 3:**
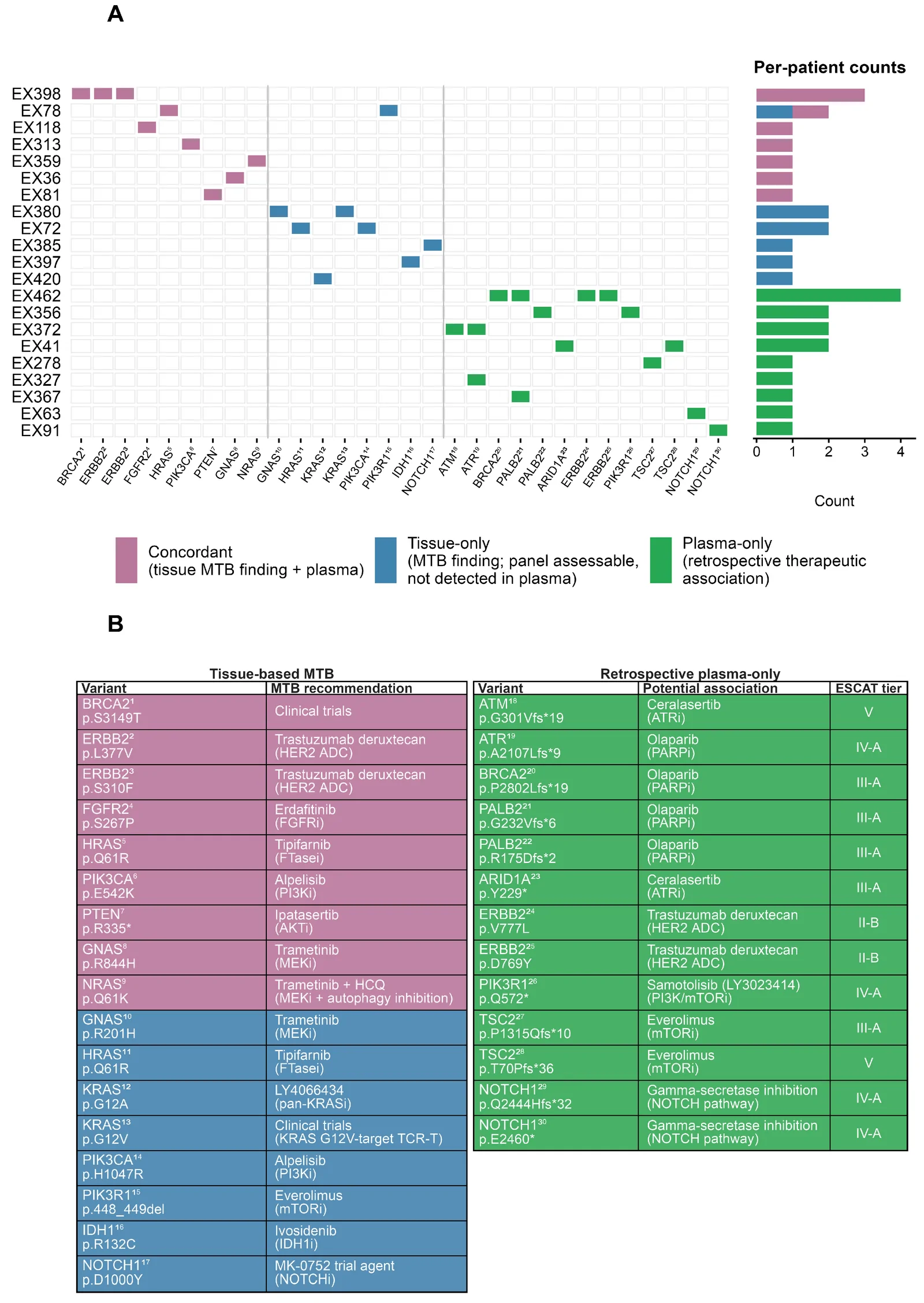
Reconciliation of tissue-based therapeutic findings and retrospectively annotated plasma-only therapeutic associations: (**A**) Patient-level comparison of variants associated with tissue-based molecular tumor board recommendations and corresponding findings from tumor-agnostic plasma profiling. Variants are classified as concordant, tissue-only, or plasma-only according to variant-level detection in matched tissue and plasma samples. Tissue-only variants indicate panel-assessable tissue variants not detected in plasma; plasma-only variants indicate retained plasma variants not detected in matched tissue. (**B**) Tissue-based therapeutic recommendations and retrospectively assigned potential therapeutic associations for plasma-only variants. Plasma-only associations were evaluated post hoc using ESCAT and are shown with their corresponding evidence tiers. These associations were assigned retrospectively for research purposes, were considered in variant, therapy, and tumor-type context, were not reviewed by the molecular tumor board, and did not guide clinical treatment decisions. Patient-level tumor entity, therapeutic rationale, evidence context, and ESCAT tier are provided in Supplementary Table S3.

**Figure 4:**
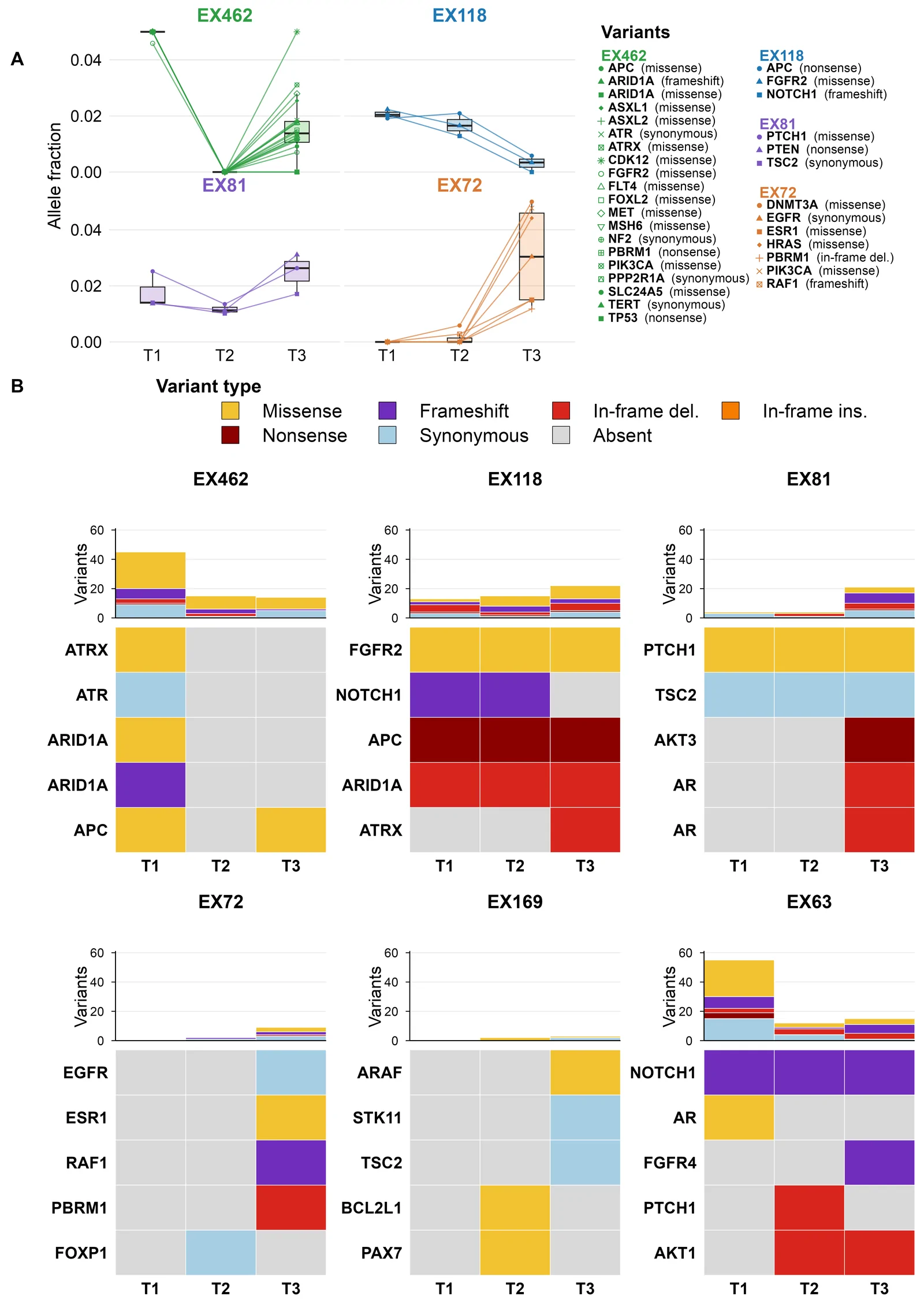
Longitudinal ctDNA dynamics under tumor-informed and tumor-agnostic analytical frameworks: (**A**) Tumor-informed tracking of tissue-concordant ctDNA variants across serial plasma samples. (**B**) Patient-level tumor-agnostic longitudinal ctDNA profiles showing variant type and per-timepoint variant counts. The top five variants by allele fraction across the longitudinal series are displayed for each patient. These longitudinal analyses are descriptive and hypothesis-generating and are not intended to establish a clinical monitoring performance or lead-time.

**Table 1:** Clinical characteristics of the EXMASTER study cohort.

Characteristic	Value
Median age (range)	48.5 years (24–78)
**Sex**	
Male	22 (55%)
Female	18 (45%)
**Disease extent**	
Metastatic disease	39 (98%)
Localized or locally advanced non-metastatic disease	1 (2%)
**Tumor entity group**	
Head and neck	17 (42%)
Colorectal	7 (18%)
Non-colorectal gastrointestinal	4 (10%)
Hepatobiliary	3 (8%)
Genitourinary	2 (5%)
Gynecologic	2 (5%)
Breast	1 (2%)
Central nervous system	1 (2%)
Lung	1 (2%)
Liver	1 (2%)
Testicular	1 (2%)

Data are presented as *n* (%) unless otherwise indicated.

## Data Availability

The data supporting the findings of this study are available from the corresponding author upon reasonable request, subject to applicable patient privacy, ethical, and institutional data-sharing restrictions.

## Supplementary Materials

The following supporting information can be downloaded at https://www.mdpi.com/article/10.3390/diagnostics16193077/s1, Figure S1: Study cohort and analytical subsets; Figure S2: Schematic overview of the end-to-end EXMASTER workflow; Figure S3: Schematic overview of baseline and longitudinal blood sampling within the EXMASTER study; Figure S4: Overview of the EXLIQUID tumor-agnostic UMI-based bioinformatic pipeline; Figure S5: Variant oncogenicity classification workflow for tumor-agnostic cfDNA analysis; Table S1: Genes included in the ExTarget targeted sequencing panel; Table S2: Baseline plasma sequencing quality metrics in the tumor-agnostic cohort; Table S3: Patient-level tumor entity, therapeutic rationale, evidence context, and retrospective ESCAT classification for therapeutically annotated findings shown in Figure 3; Table S4: Patient-level characteristics and tumor-informed variant detection in the six-patient benchmarking cohort; Table S5: Direct comparison of retained cfDNA variant calls across tumor-agnostic filtering tiers in patients with paired UMI1 and UMI3 data; Table S6: Retained tumor-agnostic plasma variants and oncogenicity classifications in the 34-patient baseline cohort; Table S7: Exploratory negative-binomial regression sensitivity analyses for Figure 2 count endpoints. References [33,34,35,36,37,38,39,40,41,42,43,44,45,46,47] are cited in Supplementary Table S3.
