# Supplementary material for: Analytical and Biological Factors Associated with Oncogenic Variant Yield in Tumor-Agnostic Plasma ctDNA Profiling
Source: Diagnostics (Basel). 2026 Sep 22;16(19):3077. doi: 10.3390/diagnostics16193077 (PMC13664594; doi:10.3390/diagnostics16193077)
Supplement: Supplementary file 1 [file diagnostics-16-03077-s001.zip › EXMASTER_Revised_Supplementary_Information.pdf]

***George S. et al.:*** **Analytical and Biological Factors Associated with Oncogenic Variant Yield in Tumor-Agnostic Plasma ctDNA Profiling**

**Supplementary Information**

*1. Sample collection and biobanking*

Peripheral blood samples were collected prospectively following informed consent using Streck Cell-Free DNA BCT® tubes (Streck, La Vista, NE, USA; Cat. No. 230245) according to a standardized EXMASTER pre-analytical workflow (Supplementary Figures S2 and S3). Baseline blood draws (T1) were performed at study inclusion and, where applicable, contemporaneously with tissue biopsy.

In a subset of patients, additional blood samples were collected longitudinally during systemic therapy as part of routine clinical follow-up. Sampling was performed at clinically relevant time points, including early on-treatment assessments and at disease progression where feasible.

Under the study SOP, the maximum permitted interval between blood collection in Streck Cell-Free DNA BCT® tubes and first centrifugation was 14 days; in practice, samples were typically processed within 7 days of collection. Exact sample-specific collection-to-first-centrifugation intervals were recorded operationally but were not treated as a prespecified study variable or included in the present analyses.

Whole blood was centrifuged at 2000 rpm for 10 minutes to separate plasma from cellular components. Plasma was transferred to a 15 ml tube, and the buffy coat fraction was collected for downstream germline and clonal hematopoiesis analyses. Plasma underwent a second centrifugation step at 2000 rpm for 10 minutes to remove residual cellular debris, followed by aliquoting into cryotubes and storage at −80 °C.

Plasma and buffy coat samples were initially biobanked at the Laboratory of Radiation Biology and Translational Radiooncology, Charité – Universitätsmedizin Berlin, and subsequently transferred to the Central Biobank of Charité (ZeBanC) for long-term storage.

Peripheral blood from healthy donors was processed using the same protocol to generate a reference exclusion dataset for downstream bioinformatic filtering. Synthetic ctDNA reference materials (Seraseq ctDNA Complete Mutation Mix, SeraCare, Milford, MA, USA; Cat. No. 0710-0532) were used for technical benchmarking at defined allele fractions (0.025, 0.01, 0.005, and wild-type control) and processed alongside patient samples.

*2. cfDNA and gDNA extraction*

cfDNA was isolated from plasma using the QIAamp Circulating Nucleic Acid Kit (Qiagen, Hilden, Germany; Cat. No. 55114) according to the manufacturer's

instructions. For each sample, 3.6 ml of plasma was used as input. cfDNA was eluted in 50 µl of elution buffer, and DNA concentration was quantified using the Qubit dsDNA High Sensitivity Assay Kit (Thermo Fisher Scientific, Waltham, MA, USA; Cat. No. Q32851).

Genomic DNA (gDNA), used for germline and clonal hematopoiesis controls, was extracted from buffy coat using the DNeasy Blood & Tissue Kit (Qiagen, Hilden, Germany; Cat. No. 69504). For each extraction, 1.8 ml of buffy coat was used as input. gDNA was eluted in 200 µl of elution buffer, and DNA concentration was measured using the Qubit dsDNA Broad Range Assay Kit (Thermo Fisher Scientific, Waltham, MA, USA; Cat. No. Q32853).

Buffy coat–derived gDNA was used to identify germline variants and to filter clonal hematopoiesis–associated alterations in downstream analyses.

### *3. Targeted sequencing panel*

cfDNA sequencing was performed using the ExTarget targeted NGS panel, developed at the DKTK EXLIQUID partner site in Freiburg. The panel encompasses 265 genes selected for relevance to solid tumor biology, therapeutic response, and resistance mechanisms and spans approximately 540 kb of genomic target space. Gene selection was based on integration of widely used commercial oncology panels and literature-curated genes associated with tumorigenesis, therapeutic response, and resistance mechanisms. A complete list of genes included in the panel is provided in Supplementary Table S1.

Genomic target regions were defined in a Browser Extensible Data (BED) file generated by the Freiburg group and implemented within the EXMASTER workflow. Custom hybrid capture probes were designed and ordered from Integrated DNA Technologies (IDT) based on this BED design.

### *4. Library preparation and sequencing*

Libraries from plasma-derived cfDNA were prepared using the IDT xGen cfDNA and FFPE DNA Library Preparation Kit (Integrated DNA Technologies, Coralville, IA, USA; Cat. No. 10010206). For each sample, 50 ng of cfDNA was used as input, followed by 9 cycles of pre-capture PCR amplification.

Libraries from buffy coat–derived genomic DNA were prepared using the IDT xGen DNA Library Prep EZ Kit (Integrated DNA Technologies, Coralville, IA, USA; Cat. No. 10009821), using 200 ng input DNA and 7 cycles of pre-capture PCR amplification.

Library concentrations were quantified using Qubit fluorometric assays (Thermo Fisher Scientific). For hybrid capture, 500 ng of each pre-capture library was enriched using the ExTarget panel. Hybridization was performed overnight (approximately 16

hours), followed by bead-based capture and washing according to the xGen Hybridization and Wash protocol. Post-capture libraries were amplified using 13 PCR cycles optimized for panel size and probe complexity.

Fragment size distributions of cfDNA and final sequencing libraries were assessed using the Agilent TapeStation system to confirm expected fragment size profiles.

Sequencing was performed in two batches corresponding to distinct analytical objectives. The initial batch comprised synthetic reference materials and matched cfDNA and buffy coat samples used for technical benchmarking and tumor-informed variant detection and was sequenced on an Illumina NextSeq 500 platform (Max Delbrück Center for Molecular Medicine, Berlin, Germany). The second batch included the expanded baseline cohort analyzed using the tumor-agnostic workflow, as well as longitudinal samples for exploratory analyses, and was sequenced on an Illumina NovaSeq 6000 platform (Institute of Pathology, Technical University of Munich (TUM), School of Medicine and Health, Munich, Germany).

Sequencing quality for the 34 baseline tumor-agnostic plasma samples was assessed using MultiQC summaries incorporating mosdepth coverage, fgbio UMI-family metrics, Picard HsMetrics and target-region metrics, Picard insert-size metrics, and samtools alignment statistics. Median target coverage was 2960× (range, 31–4629×) after consensus processing permitting UMI families of size ≥1 and 1671× (range, 11–2725×) after restricting consensus generation to UMI families of size ≥3. Picard HsMetrics reported a single usable-bases-on-target value for each baseline sample; separate UMI1- and UMI3-specific on-target percentages were not generated. Across the 34 baseline samples, median usable bases on-target was 34.6% (range, 0.4–36.7%). Median insert size was 166 bp (range, 159–178 bp). A sequencing depth of 2500× was used as a sequencing target rather than a formal exclusion threshold, and no plasma sample was excluded on the basis of sequencing coverage or other sequencing QC metrics. Plasma libraries were generated from 50 ng cfDNA input; patient-specific total cfDNA extraction yield was not systematically available. Conventional duplication rate, a separate pre-consensus raw-depth summary, and a separately defined unique-molecular-depth metric were not generated as cohort-level reporting metrics and were not retrospectively reconstructed. Sample-level QC metrics are provided in Supplementary Table S2.

*5. Bioinformatic processing*

Sequencing data were processed using UMI-aware bioinformatic workflows optimized for detection of low-frequency variants in plasma-derived cfDNA. Tumor-agnostic variant calling was performed directly from plasma sequencing data in a blinded manner, without incorporation of matched tissue information.

Analyses were conducted separately for the two sequencing batches corresponding to distinct analytical objectives.

For the initial batch used for technical benchmarking and tumor-informed detection performance, sequencing data were processed using a custom Snakemake-based pipeline. Processing steps included read demultiplexing, adapter trimming, alignment to the human reference genome (hg19, GRCh37), generation of molecular consensus reads using unique molecular identifiers (UMIs), and variant calling.

For the second sequencing batch, bioinformatic processing was performed using the EXLIQUID tumor-agnostic pipeline (see Supplementary Figure S4). Raw base call (BCL) files were converted to FASTQ format using bcl-convert, followed by adapter trimming and quality filtering. Reads were aligned using BWA-MEM to the GRCh37-based hs37d5 reference with PhiX (hs37d5_PhiX.fa) with preservation of UMI information. UMI families were grouped using fgbio to generate molecular consensus reads, enabling suppression of PCR and sequencing errors. Consensus reads were filtered to retain molecular families meeting minimum support criteria prior to downstream analysis.

Variant calling was performed at the consensus level using LoFreq. Molecular support thresholds were then applied according to the filtering configurations described below. Variant annotation and filtering, including identification of germline variants and clonal hematopoiesis–associated alterations, were performed using OpenCRAVAT and custom annotation pipelines.

Tumor-informed analyses shared the same upstream preprocessing steps, including alignment and UMI-based consensus generation. Downstream processing diverged prior to consensus-level filtering to enable targeted interrogation of variants previously identified in matched tumor tissue. Tumor-informed analyses used targeted interrogation of predefined tumor-derived variants, followed by OpenCRAVAT-based annotation to support detection and longitudinal tracking of predefined tumor-derived variants and were not restricted by a minimum UMI consensus family threshold.

For tumor-agnostic analyses, additional post-calling filtering criteria were applied to define high-confidence plasma variants, including minimum thresholds for UMI support and strand representation, as described below.

*6. Definition and evaluation of candidate tumor-agnostic filtering configurations*

Following consensus-level variant calling, three candidate filtering configurations of increasing analytical stringency were defined before inspection of the tissue-plasma concordance and retained plasma variant results. The thresholds and filtering rules defining these configurations were not subsequently modified after comparison of their performance. Tissue information was incorporated only post hoc and was not used to guide tumor-agnostic variant calling or filtering.

**Tier 1 (maximum sensitivity):** Variants supported by ≥1 UMI consensus family with minimal strand support (≥1 read on both forward and reverse strands; DP4 ≥1/1). This

configuration maximizes sensitivity and includes low-support molecular families, but is associated with higher background noise. Tier 1 was used as the denominator for calculating relative retention of tissue-concordant variants.

**Tier 2 (increased molecular confidence):** Variants supported by ≥3 independent UMI consensus families with minimal strand support (DP4 ≥1/1). This configuration reduces PCR and sequencing errors while retaining the majority of tissue-concordant variants.

**Tier 3 (highest confidence):** Variants supported by ≥3 independent UMI consensus families and strong dual-strand support (≥3 reads on both forward and reverse strands; DP4 ≥3/3). This was the most stringent of the three candidate configurations and was selected for downstream tumor-agnostic analyses after within-cohort comparison of retained plasma variant burden and tissue-concordant variant retention.

Tissue-concordant variant retention was assessed among 21 patients with panel-evaluable tissue DNA variants. Tier 1 retained 58 tissue-concordant variants, Tier 2 retained 57 (98.3% relative to Tier 1), and Tier 3 retained 53 (91.4% relative to Tier 1).

To quantify the effect of filtering stringency on the overall retained plasma call set, the direct Tier 1–3 comparison was performed in 28 patients for whom paired UMI1 and UMI3 processed datasets were available at the time of the filtering comparison. Corresponding UMI1 source data were unavailable for six of the 34 baseline patients. Across the 28 paired patients, Tier 1 retained 2,392 cfDNA variant calls, Tier 2 retained 1,070, and Tier 3 retained 501, corresponding to reductions of 55.3% and 79.1%, respectively, relative to Tier 1 (Supplementary Table S5). UMI3 data were subsequently available for the complete 34-patient tumor-agnostic baseline cohort, to which the selected Tier 3 workflow was applied. This full-cohort analysis retained 603 plasma variants. Thus, the Tier 3 count of 501 in the direct filtering comparison refers specifically to the 28-patient paired subset and is distinct from the 603 retained Tier 3 variants reported for the complete 34-patient cohort.

Tier 3 has not been independently evaluated in an external dataset.

### *7. Variant oncogenicity classification and research-oriented plasma variant interpretation*

For tumor-agnostic analyses, cfDNA variants passing consensus-level filtering were further evaluated using a structured oncogenicity classification framework to enable biologically contextualized downstream analyses (see Supplementary Figure S5). The framework was adapted from established somatic variant interpretation guidelines as described by Horak et al.[1] and aligned with molecular tumor board practices, while accounting for technical and biological constraints specific to cfDNA analysis. This framework was originally designed to support molecular tumor board–aligned variant interpretation; however, due to logistical constraints, variant classification and

interpretation for the tumor-agnostic plasma analyses were performed within the study team for research purposes and were not part of a formal molecular tumor board review process.

Variant annotation was performed using OpenCRAVAT, incorporating sequence consequence, population frequency data, cancer hotspot recurrence, functional annotations, and computational prediction scores. In addition, variant-level evidence was systematically cross-referenced with publicly available clinical and oncogenicity databases, including OncoKB (data release v6.0) and ClinVar (accessed January and February 2026, with selected variants re-reviewed in August 2026), to support evidence-based classification.

Annotated variants were harmonized across cfDNA and matched buffy coat datasets, restricted to coding regions covered by the targeted panel, and filtered to exclude off-target calls. Germline variants and clonal hematopoiesis–associated alterations were identified using matched buffy coat sequencing and gene-level annotations and were excluded from downstream analyses prior to classification. In tumor-agnostic analyses, variants in canonical clonal hematopoiesis–associated genes (*DNMT3A*, *TET2*, *ASXL1*, *JAK2*, *PPM1D*, *SF3B1*, *SRSF2*, and *U2AF1*) were excluded to further reduce non-tumor signal. *TP53* variants were not excluded *a priori* but were retained if consistent with oncogenic driver events, such as hotspot or truncating mutations, whereas low-confidence variants lacking such features were excluded.

Evidence categories were extracted for each variant and evaluated using a point-based framework adapted from Horak et al.,[1] integrating oncogenic and benign evidence to derive provisional oncogenicity classifications. Variants were assigned to standard categories, including oncogenic, likely oncogenic, variant of uncertain significance, likely benign, and benign, consistent with established somatic variant interpretation conventions. Technical flags and clonal hematopoiesis annotations were recorded to support interpretation but did not directly influence classification scores. Final study classifications were assigned after reconciliation of the provisional point-based classifications with manual variant-level review of OncoKB and ClinVar evidence. These final classifications were used to define the oncogenic or likely oncogenic variant subset for downstream analyses.

The complete retained plasma variant dataset and corresponding oncogenicity classifications are provided in Supplementary Table S6.

Filtered variants were aggregated on a per-patient basis to derive ctDNA-based endpoints, including patient-level analytical detection and retained plasma variant burden. Oncogenicity classification specifically defined the subset of retained variants classified as oncogenic or likely oncogenic and was used to derive oncogenic variant count; it was not used to guide therapeutic decision-making. These endpoints were subsequently analyzed in relation to tissue-based tumor heterogeneity metrics, including the mutant allele tumor heterogeneity (MATH) score.[2]

Potential therapeutic associations for plasma-only oncogenic or likely oncogenic variants were assigned retrospectively through targeted review of PubMed-indexed literature, ClinicalTrials.gov, and publicly accessible biomarker and variant resources, considering the specific molecular alteration, relevant therapy or drug class, and tumor type. Candidate associations were subsequently re-evaluated using the ESMO Scale for Clinical Actionability of molecular Targets (ESCAT).[3] ESCAT tiers were assigned post hoc according to the evidence available for the specific alteration–therapy–tumor context. ESCAT was used to grade the strength of evidence among retained associations rather than as a prespecified minimum-tier inclusion threshold.

For the tiers represented in this analysis, ESCAT II-B was assigned when prospective clinical trial evidence in the relevant tumor type demonstrated increased responsiveness to the matched therapy without established survival benefit; ESCAT III-A when clinical benefit for the alteration–drug match had been demonstrated in another tumor type but evidence in the patient-specific tumor type was limited or absent; ESCAT IV-A when actionability was supported by preclinical in vitro or in vivo evidence without conclusive clinical evidence; and ESCAT V when prospective studies demonstrated objective responses but without clinically meaningful outcome benefit. The ESCAT tier that best matched the available evidence for the specific alteration–therapy–tumor context was assigned.

Where evidence required cross-tumor, gene-level, pathway-level, or preclinical extrapolation, this was explicitly recorded. Associations that were not supported after formal evidence review were not retained in the final therapeutic-association analysis. The ESCAT assessment was performed by a single investigator and was not independently adjudicated. These retrospective associations were not reviewed by the molecular tumor board and did not guide clinical treatment decisions.

Patient-level information on tumor entity, therapeutic rationale, evidence context, and ESCAT tier for the therapeutically annotated tissue and plasma findings shown in Figure 3 is provided in Supplementary Table S3.

*8. Tumor heterogeneity assessment using the MATH score*

Tissue-based intratumoral genetic heterogeneity was quantified using the MATH score as originally described by Mroz and Rocco.[2] It was calculated from somatic variant allele frequencies (VAF) derived from clinical tumor sequencing performed within the DKTK MASTER program.

Somatic variant calls were obtained from tumor tissue–based Variant Call Format (VCF) files generated from clinical sequencing, which was predominantly whole-exome sequencing (WES). All somatic single nucleotide variants and small insertions and deletions passing quality filtering in the clinical pipeline were included in the MATH calculation. Variants were not restricted to genes represented on the ExTarget cfDNA panel.

For each tumor sample, the MATH score was calculated as:

**MATH = 100 × MAD (VAF) / median (VAF)**

where MAD denotes the median absolute deviation of the distribution of tumor variant allele frequencies. MATH therefore represents a normalized measure of allelic dispersion within a tumor sample.

Calculations were performed in R version 4.5.2 using standard statistical functions. MATH scores were rounded to two decimal places prior to downstream analysis.

Tissue-derived MATH reflects somatic allelic heterogeneity as captured by clinical tumor sequencing and was calculated independently of the ExTarget cfDNA panel.

*9. Definition of patient-level analytical detection and retained plasma variant burden endpoints*

ctDNA-based endpoints were defined to enable quantitative assessment of plasma molecular signal and to support downstream analyses in relation to tissue-based tumor heterogeneity.

In tumor-agnostic analyses, patient-level analytical detection was defined as the presence of at least one retained somatic plasma variant considered compatible with tumor origin after UMI-based error suppression and multi-step filtering designed to minimize technical artifacts, germline variation, clonal hematopoiesis, and recurrent background signal. This endpoint was distinct from oncogenic positivity, which required at least one retained plasma variant classified as oncogenic or likely oncogenic.

Initial variant-level quality control filters were applied to ensure sufficient sequencing quality and coverage. These included a minimum read depth of 500× at the variant position and a minimum base quality score of Phred ≥30. Variants exhibiting strong strand bias were excluded, defined as disproportionate support from a single sequencing strand. Variants located in homopolymer regions prone to sequencing artifacts were removed using a homopolymer run (HRUN) filter.

To further reduce background noise, recurrent non-tumor variants were excluded using a healthy donor–derived exclusion list generated from cfDNA samples processed and sequenced under identical conditions. Germline variants and clonal hematopoiesis–associated alterations were identified using matched buffy coat sequencing and excluded from downstream analyses.

Following these general quality control steps, stringent molecular support criteria were applied to define high-confidence plasma variants. Tumor-agnostic variant detection required support from at least three independent UMI consensus families and balanced dual-strand representation, defined as a minimum of three variant-supporting reads on both forward and reverse strands (DP4 forward ≥3 and reverse

≥3). These criteria correspond to the most stringent filtering configuration (Tier 3) as defined above and were selected for downstream analyses based on the observed within-cohort balance between reduction of the retained plasma call set and preservation of tissue-concordant variants.

Retained plasma variant burden was defined as the total number of retained plasma variants per sample after tumor-agnostic variant calling and filtering. Oncogenic variant count was defined as the number of retained plasma variants per sample classified as oncogenic or likely oncogenic according to the classification framework described above.

In tumor-informed analyses, ctDNA detection was defined as the identification of at least one tumor-derived variant previously detected in matched tumor tissue. Tumor-informed detection was evaluated independently of tumor-agnostic plasma variant burden endpoints and was used to assess sensitive detection and longitudinal tracking of predefined tumor-derived variants.

These ctDNA-based endpoints were used in exploratory analyses evaluating associations with tissue-based tumor heterogeneity metrics, including the MATH score.

**References**


1. Horak P, Griffith M, Danos AM, et al. Standards for the classification of pathogenicity of somatic variants in cancer (oncogenicity): Joint recommendations of Clinical Genome Resource (ClinGen), Cancer Genomics Consortium (CGC), and Variant Interpretation for Cancer Consortium (VICC). Genet Med. 2022;24(5):986–998.
2. Mroz EA, Rocco JW. MATH, a novel measure of intratumor genetic heterogeneity, is high in poor-outcome classes of head and neck squamous cell carcinoma. Oral Oncol. 2013;49(3):211–5.
3. Mateo J, Chakravarty D, Dienstmann R, et al. A framework to rank genomic alterations as targets for cancer precision medicine: the ESMO Scale for Clinical Actionability of molecular Targets (ESCAT). Ann Oncol. 2018;29(9):1895–1902.

**Legends for Supplementary Figures**

**Supplementary Figure S1.** Study cohort and analytical subsets. Forty patients were included. Six non-overlapping patients comprised the tumor-informed technical benchmarking cohort, while 34 underwent baseline tumor-agnostic plasma profiling. Within the baseline tumor-agnostic cohort, MATH analysis was performed in 31 patients, TMB analysis in 29 patients, and six patients underwent longitudinal analysis. Five of the six longitudinal patients had matched tumor tissue sequencing enabling tumor-informed longitudinal tracking. Analytical subsets within the baseline tumor-agnostic cohort overlap and are not mutually exclusive.

**Supplementary Figure S2.** Schematic overview of the end-to-end EXMASTER workflow. The workflow encompasses pre-analytical blood handling, plasma cfDNA extraction, targeted sequencing using the ExTarget panel, UMI-aware bioinformatic processing, research-oriented variant interpretation within a structured analytical framework, and longitudinal ctDNA monitoring.

**Supplementary Figure S3.** Schematic overview of baseline and longitudinal blood sampling within the EXMASTER study. Blood samples were collected at baseline (T1), where feasible in conjunction with tissue biopsy, and at longitudinal follow-up timepoints during systemic therapy, including early on-treatment assessments and at disease progression where feasible. T = timepoint; FU = follow-up; PD = disease progression.

**Supplementary Figure S4.** Overview of the EXLIQUID tumor-agnostic UMI-based bioinformatic pipeline. Schematic representation of the tumor-agnostic cfDNA analysis workflow applied to plasma sequencing data. Raw base call (BCL) files were converted to FASTQ format, followed by adapter trimming and quality filtering. Reads were aligned using BWA-MEM to the GRCh37-based hs37d5 reference with PhiX (hs37d5_PhiX.fa) with retention of unique molecular identifier (UMI) information. UMI families were grouped to generate molecular consensus reads, enabling suppression of PCR and sequencing errors. Consensus reads were filtered to retain molecular families meeting predefined support criteria. Variant calling was performed at the consensus level, after which predefined molecular support thresholds were applied to retain variants supported by multiple independent UMI families for downstream tumor-agnostic analysis. Detected variants were subsequently annotated and filtered using OpenCRAVAT and custom pipelines, including integration of matched buffy coat sequencing to exclude germline and clonal hematopoiesis-associated variants.

Additional filtering steps were applied to reduce technical artifacts and background noise, resulting in a high-confidence set of plasma variants for downstream analysis.

**Supplementary Figure S5.** Variant oncogenicity classification workflow for tumor-agnostic cfDNA analysis. Schematic overview of the structured variant annotation, filtering, and oncogenicity classification framework applied to cfDNA variants following consensus-level calling. Variants were annotated using OpenCRAVAT and integrated with multiple evidence sources, including sequence consequence, population frequency data, cancer hotspot recurrence, and clinical databases including OncoKB and ClinVar. Variants were harmonized with matched buffy coat sequencing to exclude germline and clonal hematopoiesis-associated alterations and restricted to coding regions covered by the targeted panel. A point-based scoring system adapted from established somatic variant interpretation guidelines was applied to derive provisional oncogenicity classifications, integrating oncogenic and benign evidence. Final study classifications additionally incorporated manual variant-level review of OncoKB and ClinVar evidence. Additional technical and contextual flags, including clonal hematopoiesis annotations, were recorded to support interpretation without directly modifying classification scores. The workflow was originally designed to support molecular tumor board-aligned interpretation; however, in this study, variant classification and interpretation were performed within the study team for research purposes and were not part of a formal molecular tumor board review process.

**Legends for Supplementary Tables**

**Supplementary Table S1.** Genes included in the ExTarget targeted sequencing panel.

**Supplementary Table S2.** Baseline plasma sequencing quality metrics in the tumor-agnostic cohort. UMI1 and UMI3 coverage values represent median target coverage after consensus processing permitting UMI families with minimum family sizes of 1 and 3, respectively, and are therefore specific to the two consensus-processing configurations. For the six longitudinal patients, TP1 was used as the baseline sample. Picard usable bases on-target represents PCT_USABLE_BASES_ON_TARGET from the available HsMetrics output, and median insert size was derived from Picard InsertSizeMetrics. These Picard metrics were available as single sample-level QC measurements and were not generated separately for UMI1 and UMI3. A sequencing depth of 2500× was used as a target rather than a formal exclusion threshold. No plasma sample was excluded on the basis of sequencing coverage or other sequencing QC metrics. EX326 was a pronounced low-coverage outlier (UMI1, 31×; UMI3, 11×) but was retained in the analysis. Excluding this sample, the lowest observed baseline coverage values were 692× for UMI1 and 308× for UMI3. Plasma libraries were generated from 50 ng cfDNA input. Patient-specific total cfDNA extraction yield was not systematically available; conventional duplication rate, a separate pre-consensus raw-depth summary, and a separately defined unique-molecular-depth metric were not generated as standardized cohort-level reporting metrics and were not retrospectively reconstructed.

**Supplementary Table S3.** Patient-level tumor entity, therapeutic rationale, evidence context, and retrospective ESCAT classification for therapeutically annotated findings shown in Figure 3. Entries 1–17 represent tissue-derived findings associated with documented molecular tumor board (MTB) recommendations; ESCAT was not reassigned to these tissue-based MTB recommendations in this post hoc analysis. Entries 18–30 represent 13 distinct plasma-only variants corresponding to 15 patient-variant/tumor contexts in 9 patients. Plasma-only therapeutic associations were assigned retrospectively for research purposes, considered in variant, therapy, and tumor-type context, and were not reviewed by the MTB or used to guide treatment. For plasma-only associations, “Therapeutic rationale” states why the drug or pathway was linked to the variant, whereas “Evidence context / source” summarizes the supporting evidence. ESCAT tiers were assigned post hoc using the published ESCAT framework and reflect the evidence available for the specific alteration–therapy–tumor

context. Cross-tumor, gene-level, pathway-level, or preclinical extrapolation is explicitly identified where applicable. The ESCAT tier should not be interpreted as a prospective clinical recommendation.

**Supplementary Table S4.** Patient-level characteristics and tumor-informed variant detection in the six-patient benchmarking cohort. Patients were selected for tumor-informed benchmarking before plasma processing because matched tumor sequencing had identified one or more variants within the ExTarget panel footprint. Selection did not depend on plasma ctDNA positivity or subsequent plasma detection results. Variant-level concordance was calculated as the number of evaluable tissue-derived variants detected in plasma divided by the total number of evaluable tissue-derived variants within the ExTarget panel footprint.

**Supplementary Table S5.** Direct comparison of retained cfDNA variant calls across tumor-agnostic filtering tiers in patients with paired UMI1 and UMI3 data. Tier 1 used the UMI1 consensus configuration with DP4 alternate-forward/reverse support ≥1/1. Tier 2 used the UMI3 consensus configuration with DP4 alternate-forward/reverse support ≥1/1. Tier 3 used the UMI3 consensus configuration with DP4 alternate-forward/reverse support ≥3/3. The direct filtering-tier comparison included 28 of the 34 tumor-agnostic baseline patients for whom paired UMI1 and UMI3 processed datasets were available. EX16, EX63, EX313, EX342, EX372, and EX397 were not included because reconstructable UMI1 data were unavailable. Tissue-concordant counts were evaluated separately among 21 patients with panel-evaluable tissue DNA variants. The Tier 3 total of 501 retained cfDNA variant calls in the 28-patient paired comparison subset is distinct from the full-cohort result; application of Tier 3 to all 34 tumor-agnostic baseline patients yielded 603 retained plasma variants.

**Supplementary Table S6.** Retained tumor-agnostic plasma variants and oncogenicity classifications in the 34-patient baseline cohort. The workbook contains an “All_variants” worksheet comprising the 603 retained plasma variants together with variant-level annotation and oncogenicity-classification information, and an “Oncogenic_variants” worksheet containing the subset of 74 retained variants classified as oncogenic or likely oncogenic. AF = allele fraction.

**Supplementary Table S7.** Exploratory negative-binomial regression sensitivity analyses for Figure 2 count endpoints. IRR = incidence rate ratio; MATH = mutant-allele tumor heterogeneity; TMB = tumor mutational burden; AF = allele fraction. Univariable negative-binomial regression models with a log link were fitted as

exploratory sensitivity analyses. Panels A and B report IRRs per 10-unit increase in MATH score, panel C per 1-unit increase in tissue TMB, and panel D per 0.001 absolute increase in median plasma AF (0.1 percentage point). Panels E and F were not modeled because panel E has a proportional outcome and panel F represents a mathematically dependent part-whole relationship. These analyses do not replace the primary descriptive Spearman analyses shown in Figure 2.
