# Supplementary material for: Analytical and Biological Factors Associated with Oncogenic Variant Yield in Tumor-Agnostic Plasma ctDNA Profiling
Source: Diagnostics (Basel). 2026 Sep 22;16(19):3077. doi: 10.3390/diagnostics16193077 (PMC13664594; doi:10.3390/diagnostics16193077)
Supplement: Supplementary file 1 [file diagnostics-16-03077-s001.zip › Supplementary_Table_S1.pdf]

Supplementary Table S1: Genes included in the ExTarget targeted sequencing panel.

ABL1	CDK4	FBXW7	IPO9-AS1	NKX2-1-AS1	RARA	TET2
ACVR2A	CDK6	FGF19	JAK2	NOMO1	RB1	TGFBR2
AKT1	CDKN1B	FGF3	JAK3	NOTCH1	REL	TIMM8B
AKT2	CDKN2A	FGF4	KDR	NOTCH2	RERGL	TMEM41A
AKT3	CDKN2B	FGFR1	KEAP1	NRAS	RET	TP53
ALK	CDKN2B-AS1	FGFR2	KIT	NTRK1	RHEB	TP63
APC	CDKN2C	FGFR3	KRAS	NTRK2	RHOA	TSC1
AR	CEBPA	FGFR4	LYRM5	NTRK3	RICTOR	TSC2
ARAF	CHEK2	FIP1L1	MAP2K1	NTRK3-AS1	RIT1	TSPAN31
ARHGEF12	CREBBP	FLT1	MAP2K2	OSMR	RNF112	U2AF1
ARID1A	CRKL	FLT3	MAP2K4	PALB2	RNF43	UGT1A1
ASXL1	CSF1R	FLT4	MAP3K1	PARP1	ROS1	UGT1A10
ASXL2	CTLA4	FOXA1	MAP3K13	PAX7	RPL21P122	UGT1A3
ATM	CTNNB1	FOXL2	MAP4K3	PBRM1	RPL21P4	UGT1A4
ATP6V0E1	CUL3	FOXO1	MAPK1	PCBP1	RPS4Y1	UGT1A5
ATR	DARC	FOXP1	MDM2	PCBP1-AS1	RUNX1	UGT1A6
ATRX	DDR2	FUBP1	MDM4	PDCD1	SDCCAG8	UGT1A7
AURKA	DDX59	GATA3	MED12	PDCD1LG2	SDHD	UGT1A8
AXL	DEFB105A	GNA11	MET	PDGFRA	SETBP1	UGT1A9
BAP1	DEFB105B	GNAQ	MFSD11	PDGFRB	SETD2	VEGFA
BCL10	DNMT3A	GNAS	MIEN1	PIK3C2G	SF3B1	VHL
BCL2	DOCK3	GNAS-AS1	MIR4728	PIK3C3	SH2D2A	YAP1
BCL2L1	DPH3	GOPC	MIR636	PIK3CA	SLC24A5	ZFY
BCR	DPYD	ADGRG6	MLH1	PIK3CB	SLC45A2	ZFY-AS1
BLOC1S6	EDAR	GTF2IP1	MPL	PIK3CD	SLC7A8	ZNF2
BRAF	EGFR	GUCY1A2	MSH2	PIK3CG	SMAD4	
BRCA1	EGFR-AS1	H3F3A	MSH6	PIK3R1	SMARCB1	
BRCA2	ELFN1	HERC2	MTOR	PIK3R2	SMIM4	
BZRAP1-AS1	EP300	HGF	MTOR-AS1	PIK3R3	SMO	
C11orf65	EPCAM	H3C2	MYC	PIP5K1A	SNAPC5	
CASP8	EPHA2	HNF1A	MYCL	PLEKHS1	SOX2	
CCND1	EPHA3	HNF1A-AS1	MYCN	PMS2	SOX2-OT	
CCND2	ERBB2	HRAS	MYCNOS	POLD1	SPEN	
CCND2-AS1	ERBB3	HSPH1	MYD88	POLE	SPOP	
CCND2-AS2	ERBB4	IDH1	NAV1	PPP2R1A	SRC	
CCND3	ESR1	IDH2	NEAT1	PTCH1	SRSF2	
CCNE1	ETV6	IGF1R	NF1	PTEN	STAG2	
CD274	EZH2	INPP4A	NF2	PTPN11	STK11	
CDH1	FAM161A	INPP4B	NFE2L2	RAC1	TBX3	
CDK12	FBXO11	INSRR	NKX2-1	RAF1	TERT	
