# Supplementary material for: Analytical and Biological Factors Associated with Oncogenic Variant Yield in Tumor-Agnostic Plasma ctDNA Profiling
Source: Diagnostics (Basel). 2026 Sep 22;16(19):3077. doi: 10.3390/diagnostics16193077 (PMC13664594; doi:10.3390/diagnostics16193077)
Supplement: Supplementary file 1 [file diagnostics-16-03077-s001.zip › Supplementary_Table_S2.pdf]

Supplementary Table S2. Baseline plasma sequencing quality metrics in the tumor-agnostic cohort.

Patient ID	UMI1 median target coverage (×)	UMI3 median target coverage (×)	Picard usable bases on-target (%)	Picard median insert size (bp)	QC note
EX16	2433	1217	34.8	165	
EX36	3806	1853	35.6	163	
EX41	3091	1796	34.1	162	
EX63	3315	1485	33.5	161	
EX69	1774	961	23.4	172	
EX72	788	308	3.6	166	
EX78	3164	1925	35.2	168	
EX81	910	812	16.6	166	
EX91	4513	2304	33.7	167	
EX118	2318	1586	35.6	165	
EX119	3402	1937	31.2	159	
EX169	692	634	20.9	170	
EX199	2581	1675	34.7	168	
EX224	3372	1899	36.7	164	
EX278	3872	2278	36.1	164	
EX313	2955	1811	36.0	166	
EX318	2319	1413	32.7	169	
**EX326**	**31**	**11**	**0.4**	**164**	Extreme low-coverage sample; retained in analysis
EX327	2318	1120	36.0	178	
EX342	3417	1707	35.5	169	
EX356	4014	2239	35.4	165	
EX357	3547	2092	36.2	169	
EX359	2610	1796	35.4	169	
EX367	2246	1037	35.4	163	
EX372	2863	1403	32.9	165	
EX380	3001	1544	32.9	171	
EX385	3437	1799	34.4	165	
EX388	3414	2260	33.6	166	
EX397	4629	2725	30.6	165	
EX398	2965	1931	35.9	169	
EX420	2734	1240	33.1	162	
EX462	3536	1667	35.5	165	
EX498	2544	1501	31.3	167	
EX515	2057	1186	34.7	167	
**Median**	**2960**	**1671**	**34.6**	**166**	
**Range**	**31–4629**	**11–2725**	**0.4–36.7**	**159–178**	

Note. Coverage values are median target coverage for baseline plasma samples. For the six longitudinal patients, time point 1 was used as the baseline sample. UMI1 denotes consensus processing permitting UMI families with a minimum family size of 1; UMI3 denotes consensus processing restricted to UMI families with a minimum family size of 3. Picard usable bases on-target corresponds to HsMetrics PCT_USABLE_BASES_ON_TARGET, defined as aligned, de-duplicated, on-target bases as a percentage of PF bases available. Picard usable bases on-target and median insert size were available as single sample-level QC measurements in the MultiQC report and were not generated separately for UMI1 and UMI3. Picard median insert size is the median insert size across all read orientations. A sequencing depth of 2500× was used as a sequencing target rather than a formal exclusion threshold. All plasma libraries were generated from 50 ng cfDNA input. No plasma sample was excluded on the basis of sequencing coverage or other sequencing-QC metrics. EX326 was a pronounced low-coverage outlier (UMI1, 31×; UMI3, 11×) but was retained in the analysis. Excluding this sample, the lowest observed baseline coverage values were 692× for UMI1 and 308× for UMI3. Patient-specific total cfDNA extraction yield was not systematically available. Conventional duplication rate, a separate pre-consensus raw-depth summary, and a separately defined unique-molecular-depth metric were not generated as cohort-level reporting metrics and were not retrospectively reconstructed.
