# Supplementary material for: Analytical and Biological Factors Associated with Oncogenic Variant Yield in Tumor-Agnostic Plasma ctDNA Profiling
Source: Diagnostics (Basel). 2026 Sep 22;16(19):3077. doi: 10.3390/diagnostics16193077 (PMC13664594; doi:10.3390/diagnostics16193077)
Supplement: Supplementary file 1 [file diagnostics-16-03077-s001.zip › Supplementary_Table_S3.pdf]

Supplementary Table S3. Patient-level tumor entity, therapeutic rationale, evidence context, and ESCAT tier for therapeutically annotated Figure 3 findings.

Fig. 3 no.	Patient ID	Tumor entity	Variant	Detection category	Therapeutic recommendation / retrospective association	Therapeutic rationale	Evidence context / source	ESCAT tier (plasma-only)
1	EX398	Colon carcinoma	BRCA2 p.S3149T	**Concordant**	Clinical trials	Documented patient-specific MTB recommendation	Study MTB record	—
2	EX398	Colon carcinoma	ERBB2 p.L377V	**Concordant**	Trastuzumab deruxtecan (HER2 ADC)	Documented patient-specific MTB recommendation	Study MTB record	—
3	EX398	Colon carcinoma	ERBB2 p.S310F	**Concordant**	Trastuzumab deruxtecan (HER2 ADC)	Documented patient-specific MTB recommendation	Study MTB record	—
4	EX118	Submandibular gland carcinoma	FGFR2 p.S267P	**Concordant**	Erdafitinib (FGFRi)	Documented patient-specific MTB recommendation	Study MTB record	—
5	EX78	Parotid gland carcinoma	HRAS p.Q61R	**Concordant**	Tipifarnib (FTasei)	Documented patient-specific MTB recommendation	Study MTB record	—
6	EX313	Stomach carcinoma	PIK3CA p.E542K	**Concordant**	Alpelisib (PI3Ki)	Documented patient-specific MTB recommendation	Study MTB record	—
7	EX81	Parotid gland carcinoma	PTEN p.R335*	**Concordant**	Ipatasertib (AKTi)	Documented patient-specific MTB recommendation	Study MTB record	—
8	EX36	Appendix carcinoma	GNAS p.R844H	**Concordant**	Trametinib (MEKi)	Documented patient-specific MTB recommendation	Study MTB record	—
9	EX359	Colon carcinoma	NRAS p.Q61K	**Concordant**	Trametinib + HCQ (MEKi + autophagy inhibition)	Documented patient-specific MTB recommendation	Study MTB record	—
10	EX380	Appendix carcinoma	GNAS p.R201H	**Tissue-only**	Trametinib (MEKi)	Documented patient-specific MTB recommendation	Study MTB record	—
11	EX72	Parotid gland carcinoma	HRAS p.Q61R	**Tissue-only**	Tipifarnib (FTasei)	Documented patient-specific MTB recommendation	Study MTB record	—
12	EX420	Urachal carcinoma	KRAS p.G12A	**Tissue-only**	LY4066434 (pan-KRASi)	Documented patient-specific MTB recommendation	Study MTB record	—
13	EX380	Appendix carcinoma	KRAS p.G12V	**Tissue-only**	Clinical trials (KRAS G12V-targeted TCR-T)	Documented patient-specific MTB recommendation	Study MTB record	—
14	EX72	Parotid gland carcinoma	PIK3CA p.H1047R	**Tissue-only**	Alpelisib (PI3Ki)	Documented patient-specific MTB recommendation	Study MTB record	—
15	EX78	Parotid gland carcinoma	PIK3R1 p.448_449del	**Tissue-only**	Everolimus (mTORi)	Documented patient-specific MTB recommendation	Study MTB record	—
16	EX397	Cheek mucosa carcinoma	IDH1 p.R132C	**Tissue-only**	Ivosidenib (IDH1i)	Documented patient-specific MTB recommendation	Study MTB record	—
17	EX385	Colon carcinoma	NOTCH1 p.D1000Y	**Tissue-only**	MK-0752 trial agent (NOTCHi)	Documented patient-specific MTB recommendation	Study MTB record	—

Supplementary Table S3 (continued). Retrospective plasma-only therapeutic associations, therapeutic rationale, evidence context, and ESCAT tier.

Fig. 3 no.	Patient ID	Tumor entity	Variant	Detection category	Therapeutic recommendation / retrospective association	Therapeutic rationale	Evidence context / source	ESCAT tier (plasma-only)
18	EX372	Parotid gland carcinoma	ATM p.G301Vfs*19	**Plasma-only**	Ceralasertib (ATRi)	Predicted ATM loss may increase reliance on ATR-mediated DNA-damage signaling, providing a rationale for ATR inhibition.	PLANETTE phase IIa (ATM-altered solid tumors): ORR 7.1% (2/28) and median PFS 3.7 months; responses were limited.[34] (PMID 42219553). The current frameshift predicts loss of function, but biallelic/protein-loss status was not established. ESCAT basis: prospective responses without clinically meaningful outcome benefit -> V.	**V**
19	EX372; EX327	Parotid gland carcinoma; Colon carcinoma	ATR p.A2107Lfs*9	**Plasma-only**	Olaparib (PARPi)	ATR truncation is predicted to impair replication-stress signaling; ATR deficiency can increase sensitivity to PARP inhibition in preclinical models.	ATR-deficient DLD-1 colorectal cancer cells were ~3-fold more sensitive to olaparib than wild-type cells.[35] (PMID 39456536). A 2026 phase II basket reported no objective responses in ATR-mutated cancers.[36] (PMID 42105598). ESCAT basis: preclinical sensitivity without supportive clinical benefit -> IV-A.	**IV-A**
20	EX462	Parotid gland carcinoma	BRCA2 p.P2802Lfs*19	**Plasma-only**	Olaparib (PARPi)	Predicted BRCA2 loss impairs homologous-recombination repair, providing a PARP-inhibitor rationale.	TAPUR phase II: BRCA1/2-altered tumors met prespecified olaparib activity criteria across multiple histologies; disease control was 41% in the histology-pooled other-solid-tumor cohort.[37] (PMID 41100773; NCT02693535). No parotid-specific efficacy was established. ESCAT basis: cross-tumor clinical benefit -> III-A.	**III-A**
21	EX462; EX367	Parotid gland carcinoma; Sertoli cell testicular carcinoma	PALB2 p.G232Vfs*6	**Plasma-only**	Olaparib (PARPi)	Predicted PALB2 loss impairs BRCA2-dependent homologous-recombination repair, providing a PARP-inhibitor rationale.	Prospective basket phase II PALB2 cohort: ORR 25% and clinical benefit rate 50% with olaparib.[36] (PMID 42105598). No parotid- or Sertoli-cell-specific efficacy was established. ESCAT basis: cross-tumor clinical benefit -> III-A.	**III-A**
22	EX356	Nasopharynx carcinoma	PALB2 p.R175Dfs*2	**Plasma-only**	Olaparib (PARPi)	Predicted PALB2 loss impairs BRCA2-dependent homologous-recombination repair, providing a PARP-inhibitor rationale.	Prospective basket phase II PALB2 cohort: ORR 25% and clinical benefit rate 50% with olaparib.[36] (PMID 42105598). No nasopharyngeal-carcinoma-specific efficacy was established. ESCAT basis: cross-tumor clinical benefit -> III-A.	**III-A**
23	EX41	Solitary fibrous tumor / hemangiopericytoma	ARID1A p.Y229*	**Plasma-only**	Ceralasertib (ATRi)	Predicted ARID1A loss may increase dependence on ATR-mediated DNA-damage responses, providing a rationale for ATR inhibition.	Prospective phase II ceralasertib monotherapy in ARID1A-deficient tumors required ARID1A loss by IHC; ORR was 14% overall, with all responses in gynecologic cancers.[38] (PMID 41686845; NCT03682289). The current truncation was not IHC-validated, and no solitary-fibrous-tumor evidence was identified. ESCAT basis: cross-tumor clinical benefit -> III-A.	**III-A**
24	EX462	Parotid gland carcinoma	ERBB2 p.V777L	**Plasma-only**	Trastuzumab deruxtecan (HER2 ADC)	ERBB2 p.V777L is an activating HER2 kinase-domain mutation, providing a target for HER2-directed therapy.	DESTINY-PanTumor01 phase II prospectively enrolled V777L as a prespecified activating HER2 mutation; the salivary gland/head-and-neck adenocarcinoma subgroup had ORR 66.7% (4/6).[39] (PMID 38710187; NCT04639219). Survival benefit for this mutation-defined subgroup is not established. ESCAT basis: same-tumor-type prospective responsiveness -> II-B. Functional V777L evidence:[40] (PMID 37272756).	**II-B**
25	EX462	Parotid gland carcinoma	ERBB2 p.D769Y	**Plasma-only**	Trastuzumab deruxtecan (HER2 ADC)	ERBB2 p.D769Y is an activating HER2 kinase-domain mutation, providing a target for HER2-directed therapy.	DESTINY-PanTumor01 phase II prospectively enrolled D769Y as a prespecified activating HER2 mutation; the salivary gland/head-and-neck adenocarcinoma subgroup had ORR 66.7% (4/6).[39] (PMID 38710187; NCT04639219). Survival benefit for this mutation-defined subgroup is not established. ESCAT basis: same-tumor-type prospective responsiveness -> II-B. Functional D769Y hyperactivity:[41] (PMID 36029027).	**II-B**
26	EX356	Nasopharynx carcinoma	PIK3R1 p.Q572*	**Plasma-only**	Samotolisib (LY3023414) (PI3K/mTORi)	PIK3R1 p.Q572* disrupts p85α-mediated PI3K inhibition, increasing PI3K activity and providing a rationale for PI3K/mTOR inhibition.	PIK3R1 Q572* disrupts p85alpha inhibitory control and hyperactivates p110alpha.[42] (PMID 31831213). Related oncogenic p85alpha mutants show inhibitor-sensitive PI3K signaling preclinically.[43] (PMID 19962665). LY3023414 phase II included PIK3R1-altered endometrial cancers (ORR 16%) but found no alteration-response correlation.[44] (PMID 31880826). ESCAT basis: preclinical actionability without biomarker-specific clinical evidence -> IV-A.	**IV-A**
27	EX278	Cholangiocarcinoma	TSC2 p.P1315Qfs*10	**Plasma-only**	Everolimus (mTORi)	Predicted TSC2 loss activates mTORC1 signaling, providing a rationale for mTOR inhibition.	A published biliary-tract-cancer actionability analysis assigned oncogenic TSC2-everolimus ESCAT III-A, reflecting clinical-benefit extrapolation from other tumor types.[45] (PMID 33754015; DOI 10.7150/thno.56539). A mutation-selected pan-cancer phase II subsequently showed limited overall activity (ORR 7%; median PFS 2.3 months).[46] (PMID 33727259). ESCAT basis: published BTC-specific III-A assignment based on cross-tumor evidence.	**III-A**
28	EX41	Solitary fibrous tumor / hemangiopericytoma	TSC2 p.T70Pfs*36	**Plasma-only**	Everolimus (mTORi)	Predicted TSC2 loss activates mTORC1 signaling, providing a rationale for mTOR inhibition.	Mutation-selected pan-cancer phase II of everolimus in TSC1/TSC2/MTOR-altered tumors: ORR 7% and median PFS 2.3 months; one TSC2 responder had biallelic inactivation and PEComa-like uterine histology.[46] (PMID 33727259). No solitary-fibrous-tumor-specific evidence was identified, and biallelic status here is unknown. ESCAT basis: prospective responses without meaningful overall benefit -> V.	**V**
29	EX63	Parotid gland carcinoma	NOTCH1 p.Q2444Hfs*32	**Plasma-only**	Gamma-secretase inhibition (NOTCH pathway)	C-terminal NOTCH1 truncation can deregulate Notch signaling, providing a rationale for pathway-directed gamma-secretase inhibition.	NOTCH PEST-domain mutations activated signaling, and Notch-mutant patient-derived xenografts were highly sensitive to the gamma-secretase inhibitor PF-03084014.[47] (PMID 25564152). No mutation-selected clinical evidence was identified for parotid carcinoma. ESCAT basis: preclinical in vivo sensitivity -> IV-A.	**IV-A**
30	EX91	Nasopharynx carcinoma	NOTCH1 p.E2460*	**Plasma-only**	Gamma-secretase inhibition (NOTCH pathway)	C-terminal PEST-region NOTCH1 truncation can deregulate Notch signaling, providing a rationale for gamma-secretase inhibition.	NOTCH PEST-domain mutations activated signaling, and Notch-mutant patient-derived xenografts were highly sensitive to the gamma-secretase inhibitor PF-03084014.[47] (PMID 25564152). No mutation-selected clinical evidence was identified for nasopharyngeal carcinoma. ESCAT basis: preclinical in vivo sensitivity -> IV-A.	**IV-A**

Note. Entries 1-17 represent tissue-derived findings associated with documented patient-specific molecular tumor board (MTB) recommendations. ESCAT was not reassigned to these tissue-based MTB recommendations in this post hoc analysis. Entries 18-30 represent 13 distinct plasma-only variants corresponding to 15 patient-variant/tumor contexts in 9 patients; entries 19 and 21 each represent a distinct variant detected in two patients. Plasma-only therapeutic associations were assigned retrospectively for research purposes, considered in variant/gene, therapy, and tumor-type context, and were not reviewed by the MTB or used to guide treatment. ESCAT tiers were assigned post hoc using the ESMO Scale for Clinical Actionability of molecular Targets (ESCAT).[33] (PMID 30137196). The tier assignment reflects the evidence available for the specific alteration-therapy-tumor context and should not be interpreted as a prospective clinical recommendation. The evidence-context/source column states the evidence feature used to assign each plasma-only ESCAT tier (same-tumor-type prospective responsiveness, cross-tumor clinical benefit, preclinical actionability, or prospective responses without clinically meaningful outcome benefit). Evidence sources are summarized in the table; cross-tumor, pathway-level, or preclinical extrapolation is stated explicitly where applicable.
