# Supplementary material for: Analytical and Biological Factors Associated with Oncogenic Variant Yield in Tumor-Agnostic Plasma ctDNA Profiling
Source: Diagnostics (Basel). 2026 Sep 22;16(19):3077. doi: 10.3390/diagnostics16193077 (PMC13664594; doi:10.3390/diagnostics16193077)
Supplement: Supplementary file 1 [file diagnostics-16-03077-s001.zip › Supplementary_Table_S4.pdf]

Supplementary Table S4. Patient-level characteristics and tumor-informed variant detection in the six-patient benchmarking cohort

Patient ID	Age (years)	Sex	Tumor type	Evaluable tissue-derived variants within ExTarget panel footprint, n	Detected in plasma, n	Variant-level concordance
EX343	44	Male	Bladder cancer	4	4	100%
EX355	39	Male	Bile duct cancer	2	2	100%
EX387	45	Male	Colon cancer	7	7	100%
EX389	36	Female	Colon cancer	10	10	100%
EX59	61	Male	Thyroid cancer	8	7	87.5%
EX62	39	Female	Bile duct cancer	2	2	100%
**Total**				**33**	**32**	**97.0%**

Note. Patients were selected for tumor-informed benchmarking before plasma processing because matched tumor sequencing had identified one or more variants within the ExTarget panel footprint. Selection did not depend on plasma ctDNA positivity or subsequent plasma detection results. Variant-level concordance was calculated as the number of evaluable tissue-derived variants detected in plasma divided by the total number of evaluable tissue-derived variants within the ExTarget panel footprint.
