# Supplementary material for: Analytical and Biological Factors Associated with Oncogenic Variant Yield in Tumor-Agnostic Plasma ctDNA Profiling
Source: Diagnostics (Basel). 2026 Sep 22;16(19):3077. doi: 10.3390/diagnostics16193077 (PMC13664594; doi:10.3390/diagnostics16193077)
Supplement: Supplementary file 1 [file diagnostics-16-03077-s001.zip › Supplementary_Table_S7.pdf]

Supplementary Table S7. Exploratory negative-binomial regression sensitivity analyses for Figure 2 count endpoints.

Panel	Association	Count outcome	Predictor and IRR scale	n	IRR (95% CI)	P value
A	Tissue-derived MATH score vs plasma-only retained variant count	Plasma-only retained variant count	MATH score, per 10-unit increase	31	0.96 (0.79–1.16)	0.644
B	Tissue-derived MATH score vs oncogenic plasma-only variant count	Oncogenic plasma-only variant count	MATH score, per 10-unit increase	31	0.90 (0.76–1.07)	0.226
C	Tissue TMB vs oncogenic variant count	Oncogenic variant count	Tissue TMB, per 1-unit increase	29	1.03 (0.92–1.15)	0.609
D	Median AF of retained plasma variants vs oncogenic variant count	Oncogenic variant count	Median AF, per 0.001 absolute increase (0.1 percentage point)	31	1.01 (0.91–1.12)	0.877

Note. IRR, incidence rate ratio; MATH, mutant-allele tumor heterogeneity; TMB, tumor mutational burden; AF, allele fraction. Univariable negative-binomial regression models with a log link were fitted as exploratory sensitivity analyses. Panels A and B report IRRs per 10-unit increase in MATH score; panel C per 1-unit increase in tissue TMB; and panel D per 0.001 absolute increase in median plasma AF (0.1 percentage point). Panels E and F were not modeled because panel E has a proportional outcome and panel F represents a mathematically dependent part-whole relationship. These analyses do not replace the primary descriptive Spearman analyses shown in Figure 2.
